# Exercise Training in Heart failure with Preserved and Reduced Ejection Fraction: A Systematic Review and Meta-Analysis

**DOI:** 10.1186/s40798-022-00464-5

**Published:** 2022-06-08

**Authors:** Jamie J. Edwards, Jamie M. O’Driscoll

**Affiliations:** 1 School of Psychology and Life Sciences, Canterbury Christ Church University, North Holmes Road, Canterbury, Kent, CT1 1QU England; 2 Department of Cardiology, St George’s University Hospitals NHS Foundation Trust, Blackshaw Road, Tooting, London, SW17 0QT England

**Keywords:** Heart failure, HFpEF, HFrEF, Exercise training

## Abstract

**Background:**

While exercise training (ET) is an established tool in heart failure (HF), no research to date has analysed the efficacy of ET in both preserved (HFpEF) and reduced (HFrEF) ejection fraction phenotypes across the same clinically important parameters.

**Methods:**

A comprehensive systematic search was performed to identify trials published between 1990 and May 2021. Controlled trials of adults reporting pre- and post-ET peak VO2, 6-min walk distance (6MWD), Minnesota Living with Heart Failure Questionnaire (MLHFQ), Kansas City Cardiomyopathy Questionnaire (KCCQ) and left ventricular ejection fraction (LVEF) were considered. Parameters of cardiac diastolic function, brain natriuretic peptides (BNP)/*N*-terminal prohormone of BNP (NTproBNP) and follow-up hospitalisation and mortality data were also analysed.

**Results:**

Ninety-three studies (11 HFpEF and 82 HFrEF) were included in the final analysis, with a pooled sample size of 11,081 participants. HFpEF analysis demonstrated significant improvements in peak VO2 (weighted mean difference: 2.333 ml·min^-1^·kg^-1^, *P*_fixed_ < 0.001), 6MWD (WMD: 35.396 m, *P*_fixed_ < 0.001), MLHFQ (WMD: − 10.932, *P*_random_ < 0.001), KCCQ (WMD: 3.709, *P*_fixed_ = 0.037) and E/e′ (WMD: − 1.709, [95% CI] = − 2.91–0.51, *P*_random_ = 0.005). HFrEF analysis demonstrated significant improvements in peak VO2 (WMD: 3.050 ml·min^-1^·kg^-1^, *P*_random_ < 0.001), 6MWD (WMD: 37.299 m, *P*_random_ < 0.001), MLHFQ (WMD: − 10.932, *P*_random_ < 0.001), LVEF (WMD: 2.677%, *P*_random_ = 0.002) and BNP/NTproBNP (SMD: − 1.349, *P*_random_ < 0.001). Outcome analysis was only performed in HFrEF, which found no significant changes in hospitalisation, all-cause mortality or composite end-points.

**Conclusion:**

ET significantly improves exercise capacity and quality of life in both HFpEF and HFrEF patients. In HFpEF patients, ET significantly improved an important index of diastolic function, with significant improvements in LVEF and NTproBNP/BNP seen in HFrEF patients only. Such benefits did not translate into significantly reduced hospitalisation or mortality after short-term follow-up.

**Graphical Abstract:**

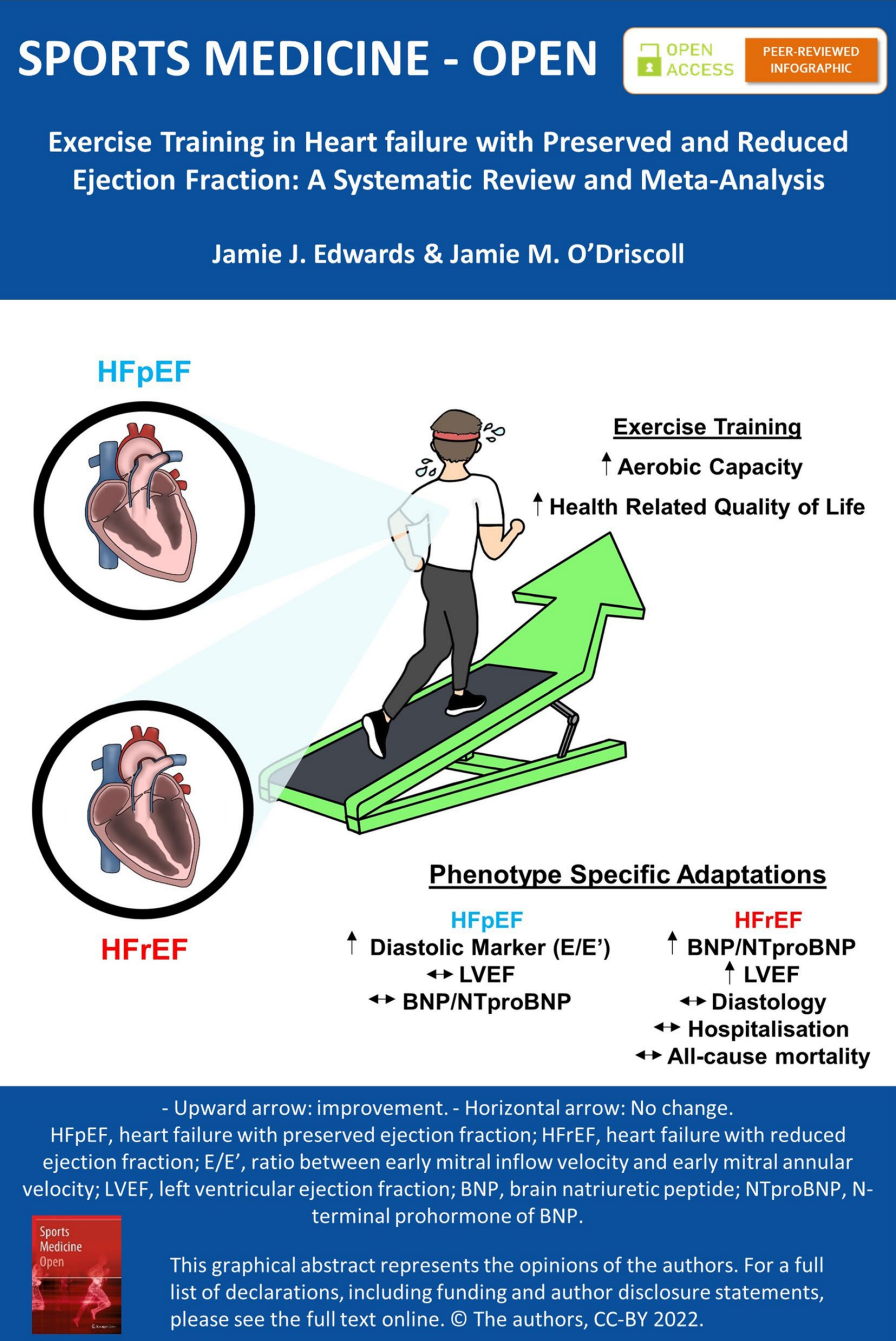

**Supplementary Information:**

The online version contains supplementary material available at 10.1186/s40798-022-00464-5.

## Key Points

- This systematic review and meta-analysis demonstrates that exercise training produces significant improvements in exercise capacity and quality of life in heart failure patients with preserved (HFpEF) and reduced (HFrEF) ejection fraction.
- While HFpEF patients observed a significant improvement in an important index of diastolic function, benefits in left ventricular ejection fraction and brain natriuretic peptides/N-terminal prohormone of BNP are only seen in HFrEF patients following exercise training.
- Although data trended in favour of exercise, such improvements did not translate into significantly reduced hospitalisation or mortality after short-term follow up.

## Introduction

Heart failure (HF) is a complex clinical syndrome, characterised by chronically insufficient cardiac systolic and/or diastolic function, attenuating the ability of the heart to effectively eject blood. Defined as a global pandemic, the epidemiological burden of HF is enormous at an estimated 64.34 million worldwide [1], or 3–20 per 1000, rising to over 100 per 1000 in those aged 65 and above [2]. HF is a leading cause of morbidity and mortality [3], carrying significant economic implications with Europe and the USA spending approximately 1–2% of their entire annual healthcare budget on HF [4, 5].

Based on various clinical characteristics, such as left ventricular ejection fraction (LVEF), brain natriuretic peptide (BNP) levels and diastolic function, HF can be dichotomised into two broad subtypes. HF with a reduced ejection fraction (HFrEF) is the traditionally recognised HF pathology, while HF with a preserved ejection fraction (HFpEF), despite having a similar prevalence to HFrEF [6, 7], is comparatively more often overlooked. While medical therapy remains central to the management of both HF pathologies, the application of adjacent non-pharmacological interventions which improve key parameters of health is crucial. Indeed, exercise training (ET) is understood as an effective tool in HF, constituting a major component of cardiac rehabilitation practices across clinics on a global scale [8, 9].

Fundamentally, there are important clinical and pathophysiological differences between HFpEF and HFrEF, which have been frequently overlooked in the ET literature. Specifically, HFpEF often presents as an elderly condition, predominantly in females preceded by chronic comorbidities such as renal insufficiency [10, 11], while HFrEF is more common in males with cardiac myocyte loss, often via familiar underlying pathology such as ischaemic heart disease [7, 12]. Despite such differences, previous ET meta-analyses have commonly analysed both subtypes as a collective [13], which conflates the results between two distinctly different pathologies, or independently [14, 15], leading to a reduced understanding of the effects of ET across the same parameters for both HFpEF and HFrEF.

As such, this systematic review and meta-analysis aims to establish the efficacy of ET in HFpEF and HFrEF across a range of important clinical parameters, including exercise capacity, quality of life, cardiac systolic and diastolic function, BNP/N-terminal proBNP and clinical outcomes from follow-up data.

## Methods

### Information Sources and Search Strategy

This systematic review and meta-analysis was performed in accordance with the PRISMA guidelines [16], with PROSPERO registration (CRD42021253793). A comprehensive computerised literature search of PubMed (Medline), the Cochrane library and Web of Science was conducted for research trials reporting the effects of an ET intervention on exercise capacity, quality of life or cardiac function in HFpEF or HFrEF. The search strategy included combinations of the relevant medical subject heading (MeSH) terms, text words and word variants for exercise, physical activity, cardiac rehabilitation, heart failure, HFpEF, HFrEF, diastolic heart failure, preserved ejection fraction and reduced ejection fraction, with the Boolean search terms “OR” and “AND” (see Appendix S1 in Additional file 1). Trials published between 1990 and May 2021 were considered. Reference lists of relevant articles and reviews were hand searched for additional reports and where relevant, corresponding authors were contacted to ascertain whether non-published data were available or in the pre-print stage.

### Study Eligibility, Outcome Measures and Data Collection

Randomised (RCT) or non-randomised (NRT) controlled trials of adults (≥ 18 years) reporting any of the following primary outcomes: peak VO_2_, 6-min walk distance (6MWD), quality of life assessed by either the Minnesota Living with Heart Failure Questionnaire (MLHFQ) [17] or the Kansas City Cardiomyopathy Questionnaire (KCCQ) [18] and/or LVEF following an exercise intervention of 4 weeks to 6 months, were considered. Secondary outcomes of interest were other markers of systolic and diastolic cardiac function including the ratio of early to late diastolic peak blood flow velocity (E/a ratio), ratio of early mitral inflow velocity and mitral annular early diastolic velocity (E/e′), left ventricular end-diastolic volume (LVEDV) and deceleration time (DT). We also acquired and analysed BNP and N-terminal proBNP (NTproBNP) (analysed as a collective via standardised means), and follow-up data of all-cause mortality, all-cause hospitalisations, HF hospitalisation and all composite endpoints (as defined in each paper). Studies without a homogenous non-intervention control group or those employing a cardiac rehabilitation intervention involving other prescribed interventions (e.g. dietary) in combination with ET were excluded. Furthermore, studies that did not provide sufficient information regarding HF subtype or analysed both HFpEF and HFrEF participants collectively were excluded, while commonly defined according to the New York Heart Association (NYHA), HFpEF and HFrEF are defined according to the respective studies individually.

The two authors (JE and JOD) independently screened all papers for eligibility. Studies were initially screened by title and abstract, and subsequently by full text if they met the relevant inclusion criteria. Any inconsistency or confliction was discussed by the researchers, and a consensus was reached. Following study recruitment, the respective data of all included studies were extracted independently by the two researchers for the analysis. If more than one study was published for the same cohort, the study containing the most comprehensive information was included to avoid overlapping populations. For those articles in which information was not adequately reported but the methodology indicates that this information would have been recorded initially, the authors were contacted.

### Study Quality Assessment

Study quality and risk of bias was evaluated using the TESTEX scale [19], which is a validated 15-point (12-item) tool designed for the specific application to exercise training studies. The two researchers independently scored all eligible articles. When disputes were detected in quality analyses, the reviewers met to discuss any conflicts and an agreement was reached. Detailed TESTEX scoring for each study can be found in Additional file 1: Table S1 and Table S2.

### Statistical Analysis

The extracted raw data were manually entered into the statistical software Comprehensive Meta-Analysis (Comprehensive Meta-Analysis Version 3, Biostat, Englewood, NJ, USA). For outcomes measured on the same scale across all studies, weighted mean difference (WMD) with 95% confidence intervals (CIs) were calculated. For outcomes measured on differing scales, standardised mean difference (SMD) with 95% CI was chosen. Outcome data were analysed separately via risk ratio (RR) with 95% CI. Pooled analyses of effect sizes were conducted separately for HFpEF and HFrEF. Further subgroup analyses of exercise type, supervised vs unsupervised training and RCT vs NRT were performed. Multiple meta-regression analyses were also conducted to ascertain if any effect moderator variables influence any of the primary outcomes. The planned moderators to be assessed independently were: age, sex, body mass index (BMI), NYHA class, study design (randomised vs non-randomised), intervention duration, HF aetiology (ischemic, dilated and hypertensive) and the baseline value of the measured primary outcome (baseline VO_2_, MLWHF and LVEF). Statistical heterogeneity was tested alongside the pooled analysis and reported as the *I*^2^ statistic. If the *I*^2^ statistic was > 40%, it was considered significant [20]. Once past this threshold, post hoc tests such as Egger’s test (1997) were systematically planned to assess the presence of funnel plot asymmetry to account for potential publication bias [21]. Random-effects analysis was selected as suggested when inter-study variability is confirmed through significant heterogeneity [20]. The results of the pooled analysis were considered significant with a *P* value of < 0.05 and a *Z*-value of > 2.

## Results

### Study and Participant Characteristics

**Fig. 1:**
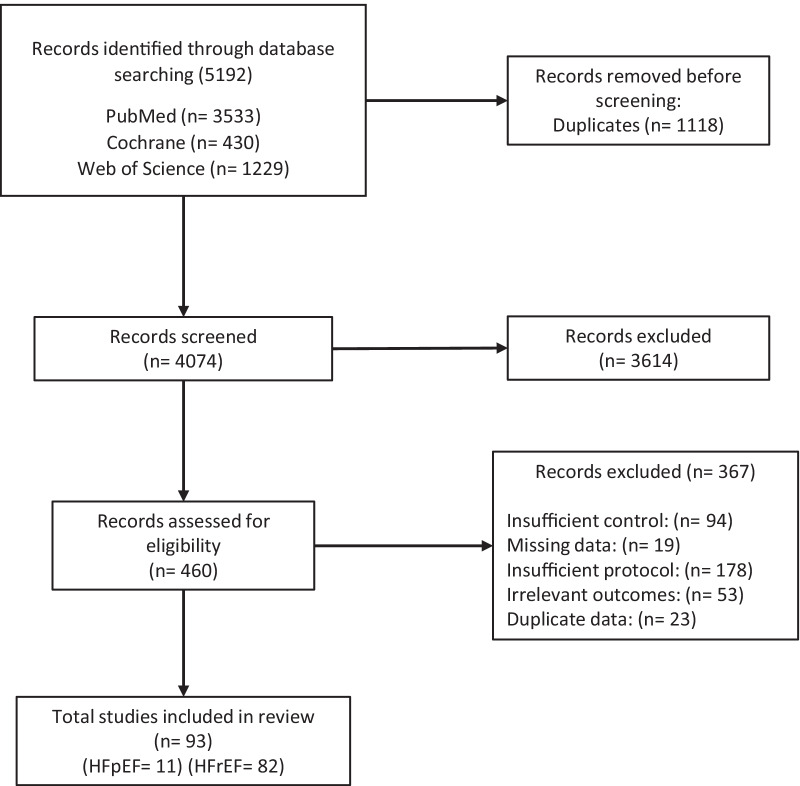
PRISMA systematic review and meta-analysis flow chart. HFpEF, Heart failure with preserved ejection fraction; HFrEF, heart failure reduced ejection fraction

Figure 1 details the PRISMA systematic review flow chart. The meta-analysis included 93 studies with a pooled sample size of 11,081 participants, which included 602 HFpEF participants and 10,479 HFrEF participants. All relevant study characteristics including TESTEX scores are presented in Tables S3 and S4 of Additional file 1 .

As detailed in Tables S3 and S4, ET appears highly safe in both HFpEF and HFrEF, with minimal acute adverse events occurring during or immediately following a training session.

### ***Exercise Capacity (Peak VO***_***2***_*** and 6MWD)***

**Fig. 2:**
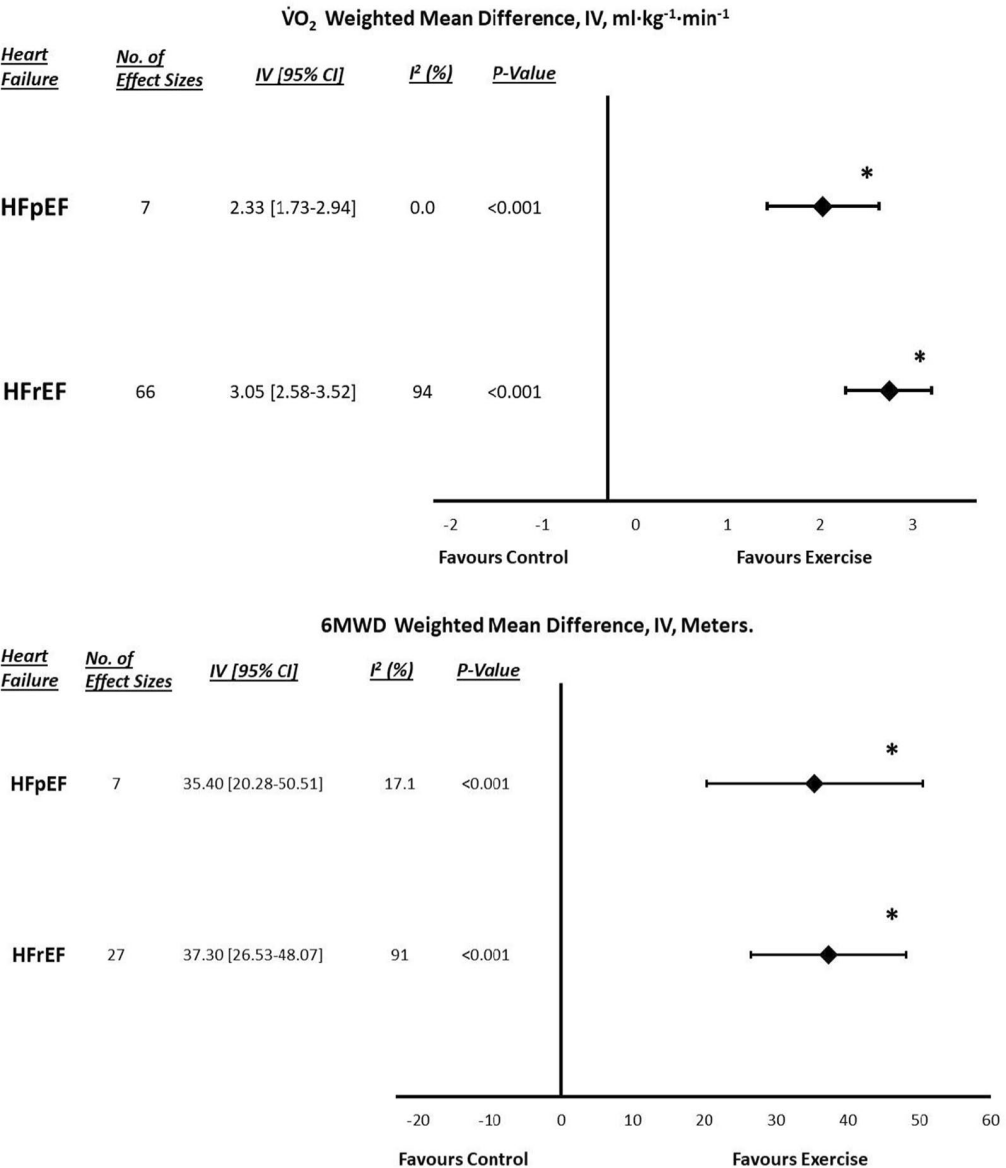
Exercise capacity (peak VO_2_ and 6MWD) forest plots for both HFpEF and HFrEF. Asterisks signify statistical significance (*P* < 0.05). HFpEF, heart failure with preserved ejection fraction; HFrEF, heart failure reduced ejection fraction; VO_2,_ volume of oxygen uptake; and 6MWD, six-minute walk distance

Figure 2 depicts the changes in exercise capacity following ET compared to the control groups. HFpEF exercise capacity analysis demonstrated a statistically significant increase in peak VO_2_ (WMD: 2.333 ml·min^-1^·kg^-1^, [95% CI] = 1.73–2.94, *P*_fixed_ < 0.001) and 6MWD (WMD: 35.396 m, [95% CI] = 20.28–50.51, *P*_fixed_ < 0.001) following ET compared to the control groups. There was no significant heterogeneity, but the post hoc Egger’s test was statistically significant (*P* < 0.001), suggesting publication bias. HFpEF meta-regression analyses showed no significant moderator effects of baseline VO_2_, age, sex, BMI, NYHA class or intervention duration (Table S5).

HFrEF exercise capacity analysis also demonstrated a statistically significant increase in peak VO_2_ (WMD: 3.050 ml·min^-1^·kg^-1^, [95% CI] = 2.58–3.52, *P*_random_ < 0.001) and 6MWD (WMD: 37.299 m, [95% CI] = 26.53–48.07, *P*_random_ < 0.001) following ET compared to the control groups. There were significant heterogeneity (*P* < 0.001, *I*^2^ = 94.0 and 91.0%) and evidence of publication bias (*p* < 0.001). HFrEF meta-regression analyses showed a significant effect of age (*B* = − 0.0955, *P* = 0.0055) and HF aetiology (*B* = 0.0972, *P* = 0.012) on exercise capacity, with younger participants and those with hypertensive HF aetiology achieving greater increases following ET (Additional file 1: Table S6, Figures S1 and S2). When dichotomised, subgroup analyses determined no significant effect of exercise type, supervision or trial design.

### Quality of Life (MLWHF and KCCQ)

**Fig. 3:**
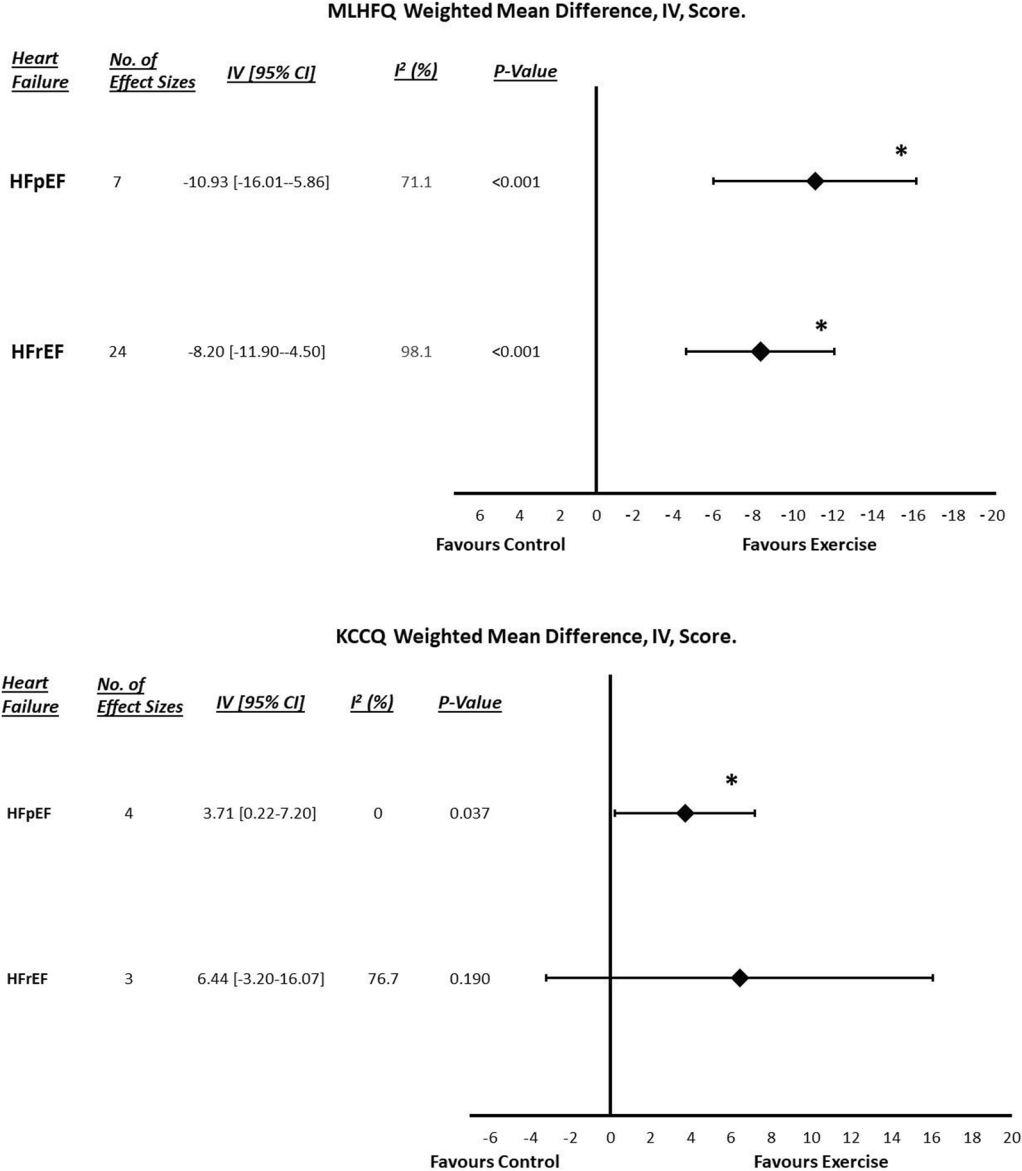
Quality of life (MLWHFQ and KCCQ) forest plots for both HFpEF and HFrEF. Asterisks signify statistical significance (*P* < 0.05). HFpEF, Heart failure with preserved ejection fraction; HFrEF, heart failure reduced ejection fraction; MLHFQ, Minnesota Living with Heart Failure Questionnaire; and KCCQ, Kansas City Cardiomyopathy Questionnaire

Figure 3 depicts the changes in quality of life following ET compared to the control groups. HFpEF quality of life analysis showed significant improvements in MLHFQ (WMD: − 10.932, [95% CI] = − 16.00–5.86, *P*_random_ < 0.001) and KCCQ (WMD: 3.709, [95% CI] = 0.22–7.12, *P*_fixed_ = 0.037) following ET compared to the control groups. There was significant heterogeneity for MLHFQ only (*P* < 0.001, *I*^2^ = 71.1%), with evidence of publication bias (*P* = 0.014). As the only statistically significant HFpEF moderator, a higher/worse baseline MLHFQ score was associated with greater improvements in quality of life following ET (*B* = − 0.7386, *P* = 0.002, Additional file 1: Table S7 and Figure S3).

HFrEF quality of life analysis also showed significant improvements in MLHFQ (WMD: − 8.199, [95% CI] = − 11.90–4.49, *P*_random_ < 0.001) following ET compared to the control groups, but there was no significant change in KCCQ (WMD: 6.436, [95% CI] = − 3.20–16.07, *P*_random_ = 0.190). There was statistically significant heterogeneity (*P* < 0.001, *I*^2^ = 98.1 and 76.7%), but no evidence of publication bias. HFrEF moderator analyses showed a higher baseline MLHFQ (*B* = − 0.2852, *P* = 0.0283) and female participant population (*B* = 0.3006, *P* = 0.0012) to be significantly associated with greater improvements in quality of life following ET (Additional file 1: Table S8, Figures S4 and S5). There were no significant subgroup effects.

### Cardiac Parameters (LVEF, E/a, E/e’, LVEDV, DT)

HFpEF cardiac function analysis determined no significant change in LVEF (WMD: 0.306%, [95% CI] = − 1.25–1.86, P_random_ = 0.699) following ET compared to the control group. Heterogeneity was high (*P* < 0.001, *I*^2^ = 76.4%), but there was no statistical significance for publication bias or the moderator analyses (Table S9). There was also no statistical significance for any other cardiac parameters, except for a significant decrease in E/e’ (WMD: -1.709, [95% CI] = − 2.91–0.51, P_random_ = 0.005) following ET in HFpEF compared to the control group.

HFrEF cardiac function analysis determined a significant increase in LVEF (WMD: 2.677%, [95% CI] = 1.01–4.34, *P*_random_ = 0.002) following ET in HFrEF compared to the control group. Heterogeneity (*P* < 0.001, *I*^2^ = 98.8%) and publication bias (*P* = 0.003) analyses were both statistically significant. Meta-regression analysis determined a significant effect of NYHA class on LVEF change, with studies containing more severe HF participants eliciting greater increases following ET (*B* = 0.0592, *P* = 0.394) (Additional file 1: Table S10 and Figure S6). There was also a significant difference between exercise type subgroups, with high-intensity interval training (HIIT) producing greater increases in LVEF compared to moderate-intensity continuous training (MICT), resistance training (RT) and combined (MICT and RT) interventions (WMD: HIIT = 11.4%, MICT = 2.2%, RT = 2.4, combined = − 0.025, *Q* = 29.175, *P* < 0.001). There was no statistical significance for any other cardiac parameters.

### BNP and NTproBNP

HFpEF analysis produced no significant change in BNP/NTproBNP following ET compared to the control group (SMD: -0.059, [95% CI] = – 0.28–0.17, *P*_fixed_ = 0.601). There was no significant heterogeneity or evidence of publication bias. There was insufficient evidence to appropriately conduct meta-regression analyses.

Conversely, HFrEF analysis produced a significant decrease in BNP/NTproBNP following ET compared to the control group (SMD: -1.349, [95% CI] =  −02.13–0.57, *P*_random_ < 0.001). There were significant heterogeneity (*P* < 0.001, *I*^2^ = 94.0%) and evidence of publication bias (*P* < 0.001). Meta-regression analyses showed a significant sex and intervention duration association, with a higher male participant population (*B* = − 0.1114, *P* = 0.040) and longer ET interventions (0.2237, *P* = 0.005) associated with a blunted reduction in BNP/NTproBNP following ET compared to the control (Additional file 1: Table S11, Figures S7 and S8).

### Follow-up (Mortality, Hospitalisation, Composite Endpoints)

**Fig. 4:**
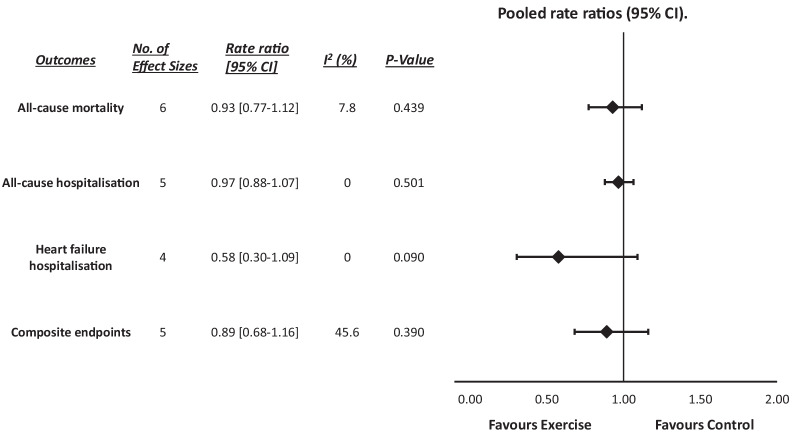
Rate ratios of all follow-up outcome data (all-cause mortality, all-cause hospitalisation, heart failure hospitalisation and composite endpoints) for both HFpEF and HFrEF. HFpEF, Heart failure with preserved ejection fraction; HFrEF, heart failure reduced ejection fraction

Figure 4 depicts the incidence ratios in the relevant outcomes following ET compared to the control groups. Pooled analysis of HFrEF follow-up data (6 months to 3 years, mean of 15.3 months) demonstrated no significant difference in incidence of all-cause mortality (RR: 0.929, [95% CI] = 0.77–1.12, *P*_fixed_ = 0.439), all-cause hospitalisation (RR: 0.967, [95% CI] = 0.88–1.07, *P*_fixed_ = 0.501), HF hospitalisation (RR: 0.576, [95% CI] = 0.30–1.09, *P*_fixed_ = 0.090) or all composite endpoints (RR: 0.890, [95% CI] = 0.68–1.16, *P*_random_ = 0.390) between the ET and control groups. None of the included HFpEF studies acquired follow-up data.

## Discussion

As the first meta-analysis to investigate both phenotypes, this study aimed to establish the efficacy of ET across various clinically relevant parameters in both HFpEF and HFrEF. Importantly, our findings show ET to be significantly effective in improving exercise capacity and quality of life in both HFpEF and HFrEF, with additional significant improvements in LVEF and BNP/NTproBNP for HFrEF only. Although data trended in favour of exercise, such improvements did not translate into significant changes in shorter-term (mean follow-up of 15 months) clinical outcomes in HFrEF patients.

Exercise capacity remains one of the strongest prognostic measures [22], with the present analysis demonstrating significant improvements in peak VO_2_ following ET by 2.33 and 3.05 ml·min-1·kg-1 for HFpEF and HFrEF, respectively, representing increases of 15.8% and 19% compared to the control groups. Previous investigations into HF cohorts have demonstrated a 6% increase in peak VO_2_ to be associated with an 8% lower risk of HF hospitalisation and a 7% lower risk of all-cause death [22], thus indicating a reduced risk of mortality following ET by 18.4% in HFpEF, and 22.2% in HFrEF. Additionally, ET produced significant increases in 6MWD of 35.4 and 37.3 m in HFpEF and HFrEF, respectively, which may also be considered clinically important [23, 24]. 6MWD is also a well-established independent predictor of clinical outcomes in HF, with increases in such magnitude associated with favourable reductions in nonfatal cardiovascular events, hospitalisations and death [25, 26]. Taken together, these exercise capacity changes are in accordance with previous work [14, 27] and carry important clinical implications, highlighting the utility of ET in both HFpEF and HFrEF.

These exercise capacity increases in HFpEF are found in the context of a significant decrease in E/e′. Indeed, previous research has clearly demonstrated E/e’ to be strongly and inversely associated with exercise capacity [28], thus implying the mechanistic contribution of an increase in diastolic function. While certainly promising for the role of ET in HFpEF diastology, it should be noted that this E/e′ improvement is somewhat driven by a study on functional electrical stimulation [29] and inspiratory muscle training [30] and thus is not entirely representative of traditional forms of ET. As demonstrated in previous meta-analyses from Pandey et al. [14] and Fukuta et al. [31], there is very little evidence to support the notion that ET improves cardiac function in HFpEF; however, the degree to which this is attributable to the limited number of published trials is unknown. Conversely, ET in HFrEF is well supported by a plethora of research trials reporting significant improvements in cardiac function and structure [15, 32, 33], with the present analysis producing a significant increase in LVEF. Similar to the diastolic change in HFpEF, this systolic improvement in HFrEF likely contributed to the observed increase in exercise capacity, while also providing its own important independent clinical implications, especially considering the lineal, inverse association between LVEF and mortality in HFrEF [34]. Of interest, there was a significant difference between exercise modes and magnitude of LVEF improvement, with HIIT producing the greatest increase by a substantial margin. While this finding certainly warrants further investigation, this analysis consists of 4 smaller-scale studies, increasing the potential for bias. It is important to note that these results contrast with those of the larger-scale SMARTEX trial [35], which reported no significant differences between HIIT and MICT, but was not included in this sub-analysis due to not including a non-intervention control group.

Although favouring exercise, the reductions in follow-up incidence rates of all-cause mortality or hospitalisation in HFrEF were non-significant. This is somewhat unsurprising since the HF-ACTION trial [36], which found no significant differences across any of these parameters unadjusted, constituted a large percentage of the population measured in this analysis. These findings echo that of the ExTraMATCH II [37] individual patient data meta-analysis of 18 trials and 3912 patients, which attributed such findings to wide CIs, potentially as a result of patient-to-patient variance in ET and usual care efficacy, as well as adherence. Nonetheless, these results are interesting given the prognostic implications of the observed exercise capacity changes. Thus, it may be hypothesised that the follow-up period of the analysed studies which represent a mean of 15 months was not long enough to see the benefits of such exercise capacity increases translate into improved clinical outcomes.

While there was a significant decrease in BNP/NTproBNP following ET in HFrEF, no such change was observed in HFpEF. These HFrEF results are consistent with most previous research trials, as exhibited in the findings of Smart et al. [38], with BNP and NTproBNP being closely associated with changes in peak VO_2_ [39]. Mechanistically, decreases in BNP and NTproBNP following ET in HF have been linked to autonomic enhancements with greater sympatho-vagal balance contributing to reduced secretion [38, 40]. While BNP and NTproBNP remain elevated irrespective of LVEF in HF, they are generally lower in HFpEF than in HFrEF with differing biomarker profiles, likely contributing to the disparity in results between the two phenotypes. Compared to that of HFrEF, the current data on the effects of ET on BNP and NTproBNP in HFpEF are limited, although to date largely indicate no clear effect. In the context of this limited data, it should be considered that BNP and NTproBNP were analysed collectively via standardised means in the present study. Although principally similar parameters, BNP and NTproBNP, are not commonly conflated in analyses, therefore this should be considered in the interpretation of these findings.

Quality of life is profoundly effected in HF and to a greater extent than that of other debilitating chronic diseases, with similar magnitudes of impairment between HFpEF and HFrEF [41, 42]. The present meta-analysis found significant improvements in quality of life following ET with MLHFQ significantly improving by 10.9 and 8.2 in HFpEF and HFrEF, respectively. KCCQ also improved for both HFpEF and HFrEF by 3.7 and 6.4, but the improvement in HFrEF was not statistically significant, likely due to broad CIs and a small sample size, highlighting the need for future research employing KCCQ measures. An improvement in MLHFQ of > 5 is considered clinically meaningful, exhibiting the efficacy of ET in both HFpEF and HFrEF in the improvement of quality of life [43]. For both HFpEF and HFrEF, greater improvements were observed in those with worse baseline quality of life scores, pointing to the need for greater urgency in the application of ET in those patients.

### Limitations

The inclusion of non-randomised trials may be perceived as a limitation of the present analysis; however, we did not want to exclude potentially valuable and clinically important data based on trial design differences. Considering the potential limitations of this, we ran subgroup and meta-regression analyses based on trial design, finding no statistically significant effects for any parameter (Additional file 1: Figures S1-S8 and Tables S5-S11). Demonstrated primarily in the HFrEF analyses, a limitation of the presented findings surrounds statistical heterogeneity. Indeed, such inter-study variance is likely, at least in part, owing to methodological differences such as study population characteristics (e.g. HF severity) and intervention diversity. Nonetheless, random-effects models and meta-regression analyses were conducted in an attempt to account for such heterogeneity. Additionally, some of the measured parameters, such as KCCQ, involved a pooled analysis of a small number of study groups, potentially impacting the reliability of drawn conclusions from such statistically powered measures. Finally, although accounted for in the meta-regression analyses, the present work collectively analysed patients of differing HF severities, thus conflating the results of patients with heterogeneous baseline characteristics.

## Conclusion

Exercise training significantly improves exercise capacity and quality of life in both HFpEF and HFrEF patients. In HFpEF patients, ET significantly improved an important index of diastolic function, with significant improvements in LVEF and NTproBNP/BNP seen in HFrEF patients only. Although data trended in favour of exercise, such improvements did not translate into significant changes in shorter-term clinical outcomes in HFrEF patients. Future research should investigate the longer-term clinical benefits of these reported adaptations in both HFpEF and HFrEF patients.

## Supplementary Information

**Additional file 1.** Search strategy details, TESTEX study scoring, tabled study characteristic data and advanced moderator analysis results.

## Data Availability

Data may be available on request to the corresponding author.
