# Supplementary material for: Exercise Training in Heart failure with Preserved and Reduced Ejection Fraction: A Systematic Review and Meta-Analysis
Source: Sports Med Open. 2022 Jun 8;8:76. doi: 10.1186/s40798-022-00464-5 (PMC9177931; doi:10.1186/s40798-022-00464-5)
Supplement: Supplementary file 1 — Additional file 1. Search strategy details, TESTEX study scoring, tabled study characteristic data and advanced moderator analysis results. [file 40798_2022_464_MOESM1_ESM.pdf]

Exercise Training in Heart failure with Preserved and Reduced Ejection Fraction: A Systematic Review and Meta-Analysis.

Jamie J. Edwards & Jamie M. O'Driscoll[1,2]

[1]School of Psychology and Life Sciences, Canterbury Christ Church University, Kent, CT1 1QU

[2]Department of Cardiology, St George's Healthcare NHS Trust, Blackshaw Road, Tooting, London, SW17 0QT.

Correspondence to Dr Jamie O'Driscoll, School of Psychology and Life Sciences, Canterbury Christ Church University, North Holmes Road, Canterbury, Kent, CT1 1 QU. Email: jamie.odriscoll@canterbury.ac.uk; Telephone: 01227782711.

Supplementary File

Appendix S1. Full systematic search

PubMed

Full search: ("exercise"[MeSH Terms] OR “exercise training”[Text Word] OR "cardiac rehabilitation"[Text Word]) AND ("heart failure"[MeSH Terms] OR "heart failure, diastolic"[MeSH Terms] OR HFpEF[Text Word] OR "preserved ejection fraction"[Text Word] OR "normal ejection fraction"[Text Word])

Cochrane

#1 MeSH descriptor: [Exercise] explode all trees

#2 MeSH descriptor: [Cardiac Rehabilitation] explode all trees

#3 #1 OR #2

#4 MeSH descriptor: [Heart Failure] explode all trees

#5 MeSH descriptor: [Heart Failure, Diastolic] explode all trees

#6 HFpEF

#7 "preserved ejection fraction"

#8 "normal ejection fraction"

Web of Science

Set 1: TS=("exercise training") OR TS=("Cardiac rehabilitation")

Set 2: TS=("heart failure") OR TS=("Diastolic heart failure") OR TS=(HFpEF) OR TS=("preserved ejection fraction") OR TS=("normal ejection fraction")

Set 3: WC=Cardiac & Cardiovascular Systems

Search Performed: #1 AND #2 AND #3 (articles only filter applied)

Table S1. Detailed HFpEF TESTEX Scoring

Study name	Eligibility criteria specified	Randomisation specified	Allocation concealment	Groups similar at baseline	Assessors blinded	Outcome measures assessed >85% of participants	Intention to treat analysis	Between group statistical comparisons reported	Point estimates reported	Activity monitoring in control group	Relative exercise intensity review	Exercise volume & energy expended	Overall TESTEX
Alves et al. (2012)	YES	YES	NO	YES	NO	YES (2)	NO	YES (2)	YES	NO	YES	NO	9
Edelmann et al. (2011)	YES	YES	NO	YES	NO	YES (2)	N/A	YES (2)	YES	YES	YES	YES	12
Fu et al. (2016)	YES	NO	NO	YES	YES	YES (1)	NO	YES (2)	YES	NO	YES	NO	8
Gary et al. (2004)	YES	YES	NO	YES	NO	YES (2)	NO	YES (2)	YES	NO	YES	NO	9
Karavidas et al. (2013)	YES	YES	NO	YES	YES	YES (2)	NO	YES (2)	YES	NO	YES	NO	10
Kitzman et al. (2010)	YES	YES	NO	YES	YES	YES (3)	N/A	YES (2)	YES	YES	YES	YES	13
Kitzman et. al (2013)	YES	YES	NO	YES	YES	YES (3)	NO	YES (2)	YES	YES	YES	YES	13
Kitzman et al. (2016)	YES	YES	NO	YES	YES	YES (2)	YES	YES (2)	YES	YES	YES	YES	13
Mueller et al. (2021)	YES	YES	YES	YES	NO	YES (2)	YES	YES (2)	YES	NO	YES	YES	12
Palau et al. (2014)	YES	YES	NO	YES	YES	YES (2)	NO	YES (2)	YES	YES	YES	YES	12
Smart et al. (2012)	YES	YES	NO	YES	NO	YES (3)	YES	YES (2)	YES	YES	YES	YES	13

Table S2. Detailed HFrEF TESTEX Scoring

Study name	Eligibility criteria specified	Randomisation specified	Allocation concealment	Groups similar at baseline	Assessors blinded	Outcome measures assessed >85% of participants	Intention to treat analysis	Between group statistical comparisons reported	Point estimates reported	Activity monitoring in control group	Relative exercise intensity review	Exercise volume & energy expended	Overall TESTEX (/15)
Acanfora et al. (2016)	YES	NO	YES	YES	YES	YES (2)	NO	YES (2)	YES	NO	YES	NO	10
Ahmad et al. (2014)	YES	YES	YES	YES	YES	YES (2)	NO	YES (2)	YES	NO	YES	NO	11
Antunes-Correa et al. (2010)	YES	N/A	NO	YES	NO	YES (2)	NO	YES (2)	YES	NO	YES	YES	9
Beckers et al. (2010)	YES	YES	NO	YES	NO	YES (1)	YES	YES (2)	YES	NO	NO	NO	8
Belardinelli et al. (1995)	YES	N/A	NO	YES	YES	YES (1)	NO	YES (2)	YES	NO	YES	YES	9
Belardinelli et al. (1999)	YES	NO	NO	YES	NO	YES (3)	YES	YES (1)	YES	NO	NO	YES	9
Belardinelli et al. (2006)	YES	NO	NO	YES	NO	YES (2)	YES	YES (2)	YES	NO	NO	NO	8
Brubaker et al. (2009)	YES	NO	NO	YES	YES	YES (2)	NO	YES (2)	YES	NO	NO	YES	9
Chien et al. (2011)	YES	NO	YES	YES	NO	YES (2)	YES	YES (2)	YES	NO	NO	NO	9
Chou et al. (2019)	YES	YES	NO	YES	YES	YES (2)	NO	YES (2)	YES	NO	YES	YES	11
Chrysohoou et al. (2015)	YES	YES	YES	YES	NO	YES (2)	YES	YES (2)	YES	NO	YES	YES	12
Conraads et al. (2004)	YES	NO	NO	YES	NO	YES (1)	NO	YES (2)	YES	NO	NO	NO	6
Corvera-Tindel et al. (2004)	YES	NO	NO	YES	NO	YES (2)	YES	YES (2)	YES	YES	YES	YES	11
Dalal et al. (2019)	YES	YES	YES	YES	YES	YES (2)	YES	YES (2)	YES	NO	YES	NO	12
de Mello Franco et al. (2006)	YES	NO	NO	YES	NO	YES (1)	NO	YES (2)	YES	NO	YES	YES	8
Demopoulos et al. (1997)	YES	N/A	NO	YES	NO	NO	NO	YES (2)	YES	NO	YES	YES	7
Dracup et al. (2007)	YES	YES	NO	YES	YES	YES(1)	YES	YES (2)	YES	YES	YES	YES	11
Du et al. (2018)	YES	YES	NO	YES	YES	YES (3)	YES	YES (2)	YES	NO	YES	NO	12
Eleuteri et al. (2013)	YES	NO	NO	YES	NO	YES (3)	YES	YES (2)	YES	NO	YES	YES	11

Erbs et al. (2010)	YES	YES	YES	YES	YES	YES (3)	NO	YES (2)	YES	NO	NO	NO	11
Fayazi et al. (2013)	YES	N/A	NO	YES	NO	YES (2)	NO	YES (2)	YES	NO	NO	NO	7
Flynn et al. (2009)	YES	YES	YES	YES	YES	YES (2)	NO	YES (2)	YES	NO	YES	NO	11
Fraga et al. (2007)	YES	NO	NO	YES	YES	NO	NO	YES (2)	YES	YES	YES	YES	9
Freimark et al. (2007)	YES	N/A	NO	YES	NO	YES (1)	NO	YES (2)	YES	NO	YES	YES	8
Fu et al. (2016)	YES	NO	NO	YES	YES	YES (2)	NO	YES (2)	YES	NO	YES	YES	10
Gademan et al. (2008)	YES	N/A	NO	YES	NO	NO	NO	YES (2)	YES	NO	YES	YES	7
Gary et al. (2012)	YES	YES	YES	YES	NO	YES (2)	NO	YES (2)	YES	NO	YES	YES	11
Giannuzzi et al. (2003)	YES	NO	NO	YES	NO	YES (2)	YES	YES (2)	YES	NO	YES	YES	10
Gielen et al. (2003)	YES	NO	NO	YES	YES	YES (1)	NO	YES (2)	YES	NO	YES	YES	9
Gottlieb et al. (1999)	YES	NO	NO	YES	NO	YES (1)	NO	YES (2)	YES	YES	YES	NO	8
Groehs et al. (2015)	YES	NO	NO	YES	YES	NO	NO	YES (2)	YES	YES	YES	YES	9
Groennebaek et al. (2019)	YES	YES	NO	YES	YES	YES (3)	NO	YES (2)	YES	NO	NO	NO	10
Hambrecht et al. (1998)	YES	NO	NO	YES	NO	YES (2)	NO	YES (2)	YES	NO	NO	YES	8
Hambrecht et al. (2000)	YES	YES	NO	YES	NO	YES (3)	NO	YES (2)	YES	NO	YES	YES	11
Harjola et al. (2006)	YES	NO	NO	YES	NO	YES (1)	NO	YES (2)	YES	NO	YES	YES	8
Höllriege et al. (2016)	YES	YES	NO	YES	YES	YES (2)	NO	YES (2)	YES	NO	YES	NO	10
Huang et al. (2014)	YES	N/A	NO	YES	NO	YES (1)	NO	YES (2)	YES	NO	YES	YES	8
Isaksen et al. (2015)	YES	NO	NO	YES	YES	YES (3)	NO	YES (2)	YES	NO	NO	NO	9
Jolly et al. (2009)	YES	YES	NO	YES	NO	YES (2)	YES	YES (2)	YES	NO	YES	NO	10
Kemps et al. (2010)	YES	N/A	NO	YES	NO	YES (3)	NO	YES (2)	YES	NO	YES	YES	10
Kiilavuori et al. (1996)	YES	NO	NO	YES	NO	YES (1)	NO	YES (2)	YES	NO	YES	YES	8
Kobayashi et al. (2003)	YES	NO	NO	YES	NO	YES (3)	YES	YES (2)	YES	NO	NO	NO	9
Koukouvou et al. (2004)	YES	YES	NO	YES	NO	YES (2)	YES	YES (2)	YES	NO	YES	YES	11

Lan et al. (2020)	YES	NO	NO	YES	YES	YES (2)	NO	YES (2)	YES	NO	YES	YES	10
Maiorana et al. (2011)	YES	YES	NO	YES	NO	Yes (2)	No	YES (2)	YES	NO	YES	YES	10
Mandic et al. (2009)	YES	YES	NO	YES	YES	YES (2)	YES	YES(2)	YES	NO	NO	YES	11
McKelvie et al. (2002)	YES	YES	NO	YES	YES	YES (2)	NO	YES (2)	YES	NO	YES	YES	11
Meirelles et al. (2014)	YES	NO	NO	YES	NO	NO	NO	YES (2)	YES	NO	YES	YES	7
Myers et al. (2007)	YES	NO	NO	YES	YES	YES (1)	NO	YES (2)	YES	NO	YES	NO	8
Nishi et al. (2011)	YES	NO	NO	YES	NO	YES (2)	NO	YES (2)	YES	NO	NO	NO	7
Nobre et al. (2016)	YES	NO	NO	YES	YES	YES (2)	NO	YES (2)	YES	NO	YES	YES	10
O'Connor et al. (2009)	YES	YES	YES	YES	YES	YES (3)	YES	YES (2)	YES	YES	NO	YES	14
Oka et al. (2000)	YES	YES	NO	YES	NO	YES (3)	NO	NO	YES	YES	NO	YES	9
Palevo et al. (2009)	YES	YES	NO	YES	NO	YES (3)	NO	NO	YES	NO	YES	YES	9
Parnell et al. (2002)	YES	YES	NO	YES	YES	YES (2)	YES	YES (2)	YES	NO	YES	NO	11
Parnell et al. (2005)	YES	NO	NO	YES	NO	YES (1)	NO	YES (2)	YES	NO	YES	NO	7
Piotrowicz et al. (2020)	YES	YES	YES	YES	YES	YES (3)	YES	YES (2)	YES	YES	YES	NO	14
Pozehl et al. (2010)	YES	YES	YES	YES	NO	YES (2)	NO	YES (2)	YES	NO	YES	YES	11
Ricca-Mallada et al. (2017)	YES	NO	NO	YES	YES	YES (2)	NO	YES (2)	YES	NO	YES	YES	10
Roveda et al. (2003)	YES	NO	NO	YES	YES	YES (3)	NO	YES (2)	YES	NO	YES	YES	11
Safiyari-Hafizi et al. (2016)	YES	NO	NO	YES	YES	YES (2)	NO	YES (2)	YES	NO	YES	YES	10
Sandri et al. (2012)	YES	YES	NO	YES	NO	YES (3)	YES	YES (2)	YES	NO	NO	NO	10
Sarullo et al. (2006)	YES	YES	YES	YES	YES	YES (3)	YES	YES	YES (2)	NO	NO	YES	13
Selig et al. (2004)	YES	YES	YES	YES	NO	YES (2)	NO	YES (2)	YES	YES	YES	YES	12
Senden et al. (2005)	YES	NO	NO	YES	NO	YES (2)	NO	YES (2)	YES	NO	YES	YES	9
Servantes et al. (2012)	YES	YES	NO	YES	NO	YES (2)	YES	YES	YES (2)	YES	YES	YES	12
Servantes et al. (2018)	YES	YES	NO	YES	YES	YES (2)	YES	YES (2)	YES	NO	YES	NO	11

Spee et al. (2016)	YES	YES	YES	YES	YES	YES (2)	NO	YES (2)	YES	NO	NO	YES	11
Stevens et al. (2015)	YES	NO	NO	YES	NO	YES (2)	NO	YES (2)	YES	NO	NO	NO	7
Sturm et al. (1999)	YES	YES	NO	YES	YES	YES (2)	NO	YES (2)	YES	NO	YES	YES	11
Tasoulis et al. (2010)	YES	NO	NO	YES	NO	YES (1)	NO	YES (2)	YES	NO	YES	YES	8
Testa et al. (2000)	YES	N/A	NO	YES	NO	NO	NO	YES (2)	YES	NO	NO	NO	5
Tyni-Lenné et al. (1996)	YES	NO	NO	YES	NO	YES (2)	NO	YES (2)	YES	NO	NO	YES	8
Tyni-Lenné et al. (2001)	YES	YES	NO	YES	NO	YES (2)	YES	YES	YES (2)	NO	YES	YES	11
Van Berendoncks et al. (2010)	YES	N/A	NO	YES	YES	YES (1)	NO	YES (2)	YES	NO	YES	YES	9
van den Berg-Emons et al. (2004)	YES	YES	NO	NO	YES	YES (1)	NO	YES (2)	YES	NO	NO	NO	7
Willenheimer et al. (1998)	YES	NO	NO	YES	YES	YES (2)	YES	YES (2)	YES	NO	NO	YES	10
Wisløff et al. (2007)	YES	YES	NO	YES	NO	YES(3)	YES	YES	YES (2)	NO	YES	YES	12
Witham et al. (2005)	YES	YES	NO	YES	YES	YES (3)	YES	YES (2)	YES	NO	YES	NO	12
Witham et al. (2012)	YES	YES	NO	YES	YES	YES (3)	YES	YES (2)	YES	NO	YES	NO	12
Yeh et al. (2011)	YES	YES	YES	YES	YES	YES (3)	YES	YES (2)	YES	NO	NO	NO	12
Zurek et al. (2012)	YES	N/A	NO	YES	NO	NO	NO	YES (2)	YES	NO	NO	NO	5

Table S3. HFpEF Study Characteristics

Study	Country	Trial Design	N. of Participants	ET type	ET Intensity	ET Frequency (per week)	ET Intervention Duration	Adverse Events	TESTEX Score
Alves et al. (2012)	Israel	RCT	31	Aerobic (cycling)	70-75% MHR	3	24 weeks	0	9
Edelmann et al. (2011)	Germany	RCT	64	Aerobic + Resistance training	Aerobic: 40-50% HRR up to 70% HRR Resistance: 65% 1RM	3	12 weeks	0 (11 without clinical relevance)	12
Fu et al. (2016)	Taiwan	NRT	60	Aerobic interval training (cycling ergometry)	80% peak VO2	3	12 weeks	NR	8
Gary et al. (2004)	USA	RCT	32	Walking	40% target HR	3	12 weeks	0	9
Karavidas et al. (2013)	Greece	RCT	30	Functional electrical stimulation	25Hz (adjusted to achieve a visible contraction)	5	6 weeks	NR	10
Kitzman et al. (2010)	USA	RCT	46	Aerobic (walking and cycling ergometry)	40-50% HRR up to 70% HRR	3	16 weeks	0	13
Kitzman et. al (2013)	USA	RCT	63	Aerobic (walking and cycling ergometry)	40-50% HRR up to 70% HRR	3	16 weeks	0 (1 patient developed transient hypoglycaemia)	13
Kitzman et al. (2016)	USA	RCT	49	Walking	20 mins-intensity NR	3	20 weeks	2	13
Mueller et al. (2021)	Germany	RCT	178	HIIT (4x4-min intervals w 3 min recovery, cycling)	80-90% HRR	3	12 weeks	36	12
				Aerobic (cycling)	35-50% HRR	5		39	
Palau et al. (2014)	Spain	RCT	26	Inspiratory muscle strength test	25-30% max inspiratory mouth pressure	Twice daily	12 weeks	NR	12
Smart et al. (2012)	Australia	RCT	25	Aerobic (cycling)	60-70% peak VO2 (30 mins)	3	16 weeks	0	13

HIIT= High-intensity interval training, HRR= Heart rate reserve, MHR= Maximal heart rate, 1-RM= 1 repetition maximum.

Table S4. HFrEF Study Characteristics.

Study	Country	Trial Design	N. of Participants	ET type	ET Intensity	ET Frequency (per week)	ET Intervention Duration	Adverse Events to ET	TESTEX Score
Corvera-Tindel et al. (2004)	USA	RCT	79	Aerobic (walking)	65% MHR	5	12 weeks	0	11
Belardinelli et al. (1995)	Italy	NRT	27	Aerobic (cycling)	40% Peak VO2	3	8 weeks	0	9
Fu et al. (2016)	Taiwan	NRT	60	Interval training (cycling)	80% Peak VO2	3	12 weeks	NR	10
Belardinelli et al. (1999)	Italy	RCT	99	Aerobic (cycling)	60% Peak VO2	3	8 weeks	0	9
Acanfora et al. (2016)	Italy	RCT	61	Aerobic (cycling), strength and flexibility exercise	60-85% Peak VO2	5	4 weeks	0	10
Meirelles et al. (2014)	Brazil	RCT	30	Aerobic (treadmill running), resistance and stretching	5-15% above VT	3	6 months	NR	7
Belardinelli et al. (2006)	Italy	RCT	52	Aerobic (cycling)	60% Peak VO2	3	8 weeks	0	8
Jolly et al. (2009)	UK	RCT	169	Aerobic (walking) and resistance	70% peak VO2	3-5	6 months	NR	10
McKelvie et al. (2002)	Canada	RCT	181	Aerobic (cycling, treadmill and arm ergometry) and resistance	60-70% MHR	3	3 months	0	11
Lan et al. (2020)	Australia	RCT	36	Aerobic (cycling and walking)	50-70% peak VO2	NR	12 weeks	NR	10
				Resistance training (dynamic and circuit)	50-70% 1-RM				
de Mello Franco et al. (2006)	Brazil	RCT	29	Aerobic (cycling)	Up to 10% below RCP	3	4 months	NR	8
Palevo et al. (2009)	USA	RCT	16	Resistance training (dynamic)	60% 1RM	3	8 weeks	0	9
Kobayashi et al. (2003)	Japan	RCT	28	Aerobic (cycling)	HR equivalent of VT	2-3	3 months	0	9
Willenheimer et al. (1998)	Sweden	RCT	49	Interval training (cycling)	80% peak VO2	3	16 weeks	0	10
Selig et al. (2004)	Australia	RCT	39	Resistance exercise	‘Moderate'	3	3 months	0	12
Erbs et al. (2010)	Germany	RCT	37	Aerobic (cycling)	50% peak VO2	Encouraged to perform daily (1 supervised)	12 weeks	NR	11

Gielen et al. (2003)	Germany	RCT	20	Aerobic (cycling and walking), calisthenics and non-competitive ball games	70% peak VO2	Encouraged to perform daily (1 supervised)	6 months	0	9
Hambrecht et al. (2000)	Germany	RCT	64	Aerobic (cycling and walking), calisthenics and non-competitive ball games	70% peak VO2	Encouraged to perform daily (1 supervised)	6 months	NR	11
Beckers et al. (2010)	Belgium	RCT	69	Gymnastics and Aerobic (cycling) (Home-based)	90% HR achieved at AT	3	6 months	NR	8
				Aerobic (cycling, treadmill etc) and resistance training (dynamic weightlifting) (Supervised ET)	90% HR achieved at AT and 50% 1-RM	3			
				Any (participant choice of training)	Any	Any			
Harjola et al. (2006)	Finland	RCT	17	Aerobic (cycling)	50-60% peak VO2	3	3 months	NR	8
Pozehl et al. (2010)	USA	RCT	42	Aerobic and resistance combined	40-70% HRR	3 aerobic, 2 resistance	12 weeks	NR	11
Stevens et al. (2015)	Belgium	RCT	22	Aerobic and resistance combined	Second VT HR and 50-70% 1RM	2-3	12 weeks	0	7
Safiyari-Hafizi et al. (2016)	Canada	RCT	40	Aerobic (walking)	80-85% peak VO2	3	12 weeks	0	10
Nishi et al. (2011)	Japan	Retrospective (NRT)	36	Aerobic (walking, cycling, calisthenics)	30-50% HRR	5	3 months	NR	7
Giannuzzi et al. (2003)	Italy	RCT	89	Aerobic (cycling)	60 peak VO2	3-5	6 months	0	10
Roveda et al. (2003)	Brazil	RCT	16	Aerobic (cycling)	HR corresponding to AT	3	4 months	0	11
Antunes-Correa et al. (2010)	Brazil	NRT	40	Aerobic (cycling) (Male)	HR corresponding to AT	3	4 months	NR	9
Demopoulos et al. (1997)	USA	NRT	23	Aerobic (cycling)	50% peak VO2	4	12 weeks	NR	7
Groehs et al. (2015)	Brazil	RCT	26	Aerobic (cycling)	HR corresponding to AT	3	4 months	NR	9
Freimark et al. (2007)	Israel	NRT	56	Aerobic (treadmill, cycling and stair machine)	60-70% MHR	2	18 weeks	NR	8
Isaksen et al. (2015)	Norway	RCT	35	Aerobic interval training	4 min intervals of 85% peak HR	3	12 weeks	0 (1 SVT during baseline test)	9
Gademan et al. (2008)	Netherlands	NRT	34	Aerobic (cycling)	HR corresponding to AT	2-3	4 weeks	NR	7
Nobre et al. (2016)	Brazil	RCT	55	Aerobic (treadmill walking)	HR corresponding to AT	3	4 months	NR	10
Chrysohoou et al. (2015)	Greece	RCT	72	High intensity intermittent aerobic training (cycling) and resistance exercise (dynamic)	80-100% Wrpeak and 30, 50, then 90%1RM	3	12 weeks	0	12

Oka et al. (2000)	USA	RCT	24	Aerobic (walking) and resistance exercise combined	70% MHR and 75% 1-RM	3	3 months	0	9
Conraads et al. (2004)	Belgium	NRT	49	Aerobic and resistance combined	90% ventil threshold and 50-60% 1-RM	3	4 months	NR	6
Kiilavuori et al. (1996)	Finland	RCT	27	Aerobic (walking, cycling, rowing swimming)	50-60% peak VO2	3	6 months	NR	8
Groennebaek et al. (2019)	Denmark	RCT	36	Blood flow restricted resistance exercise	30% 1-RM	3	6 weeks	0	10
				Remote ischemic conditioning (upper arm)	4 cycles of 5-min				
Van Berendoncks et al. (2010)	Belgium	NRT	80	Aerobic-only or combined aerobic and resistance	90% HR achieved at AT and 50% 1-RM	3	4 months	NR	9
Parnell et al. (2002)	Australia	RCT	21	Aerobic (walking, light hand weights and stationary cycling)	50-60% MHR	3-7	8 weeks	NR	11
Gary et al. (2012)	USA	RCT	24	Combined aerobic and resistance exercise	50-70% HRR and <15 RPE	3 (both)	12 weeks	0	11
Ricca-Mallada et al. (2017)	Uruguay	RCT	34	Aerobic (cycling or treadmill)	50-80% MHR	3	24 weeks	0	10
Chou et al. (2019)	Taiwan	RCT	34	HIIT (cycling)	5x3-min intervals at 80% peak VO2	3	12 weeks	NR	11
Spee et al. (2016)	Netherlands	RCT	26	HIIT (cycling)	85-95% peak VO2	3	12 weeks	0	11
Dracup et al. (2007)	USA	RCT	173	Combined aerobic (walking and resistance)	40-60% MHR and 80% 1-RM	4	6 months	NR	11
Maiorana et al. (2011)	Australia	RCT	36	Aerobic (cycling and walking)	50-70% Peak VO2	3	12 weeks	NR	10
				Resistance (dynamic weight training)	50-70%1RM				
Brubaker et al. (2009)	USA	RCT	59	Aerobic (walking and cycling)	40-70% HRR	3	16 weeks	NR	9
Du et al. (2018)	Australia	RCT	132	Walking (mirror of the 6MWT)	6MWT parallel protocol)	1	6 months	0	12
Eleuteri et al. (2013)	Italy	RCT	21	Aerobic (cycling)	HR corresponding to VT	5	3 months	0	11
Kemps et al. (2010)	Netherlands	NRT	48	Interval cycling, muscle resistance and inspiratory muscle training	50% max short-term exercise capacity, 70% 1-RM, 30% max inspiratory pressure.	3	12 weeks	NR	10
Chien et al. (2011)	Taiwan	RCT	51	Aerobic (walking) combined with strength training	NR	3	8 weeks	0	9
Tyni-Lenné et al. (1996)	Sweden	RCT	21	Double leg knee extensor endurance exercise	60 reps/min, 70% peak performance	3	8 weeks	NR	8
				Single leg knee extensor endurance exercise					
Senden et al. (2005)	Netherlands	RCT	61	Flexibility, strength and endurance training	50% Max short-term exercise capacity	4	26 weeks	0	9

Fraga et al. (2007)	Brazil	RCT	27	Aerobic (cycling)	Up to 10% below RCP	3	4 months	NR	9
Hambrecht et al. (1998)	Germany	RCT	20	Aerobic (cycling)	70% HR Peak VO2	5	6 months	0	8
Fayazi et al. (2013)	Iran	NRT	60	Aerobic (walking)	NR	3	8 weeks	0	7
Zurek et al. (2012)	Switzerland and Italy	NRT	96	Aerobic (cycling) and calisthenic	60-80% peak VO2	3	3 months	NR	5
Sturm et al. (1999)	Austria	RCT	26	Step aerobics and cycling	50% peak VO2	2-3	12 weeks	NR	11
Sarullo et al. (2006)	Italy	RCT	60	Aerobic (cycling)	60-70% peak VO2	3	3 months	0 (6 had PVCs)	13
Tyni-Lenné et al. (2001)	Sweden	RCT	24	Resistance band exercise	13-16 RPE	3	8 weeks	0	11
Wisløff et al. (2007)	Norway	RCT	27	Aerobic (uphill walking)	70-75% peak HR	3	12 weeks	0	12
				Aerobic interval training (uphill walking)	4x4-min intervals at 90-95% peak HR				
Witham et al. (2005)	UK	RCT	82	Seated exercise movements	RPE 11-13	2-3	6 months	0	12
van den Berg-Emons et al. (2004)	Netherlands	RCT	34	Aerobic (cycling, walking and games)	60% of HRR	2	3 months	NR	7
Höllriege et al. (2016)	Germany	RCT	37	Aerobic (cycling)	50%-60% symptom-limited peak VO2	3-6	12 months	N/A	10
Servantes et al. (2012)	Brazil	RCT	50	Aerobic (walking)	HR corresponding to AT	3-4	3 months	0	12
				Aerobic and strength training	30-40% 1-RM				
Yeh et al. (2011)	USA	RCT	100	Tai Chi	No metric applied	2	12 weeks	0	12
Testa et al. (2000)	USA	NRT	15	Aerobic (cycling)	50% peak VO2	4	12 weeks	NR	5
Gottlieb et al. (1999)	USA	RCT	25	Aerobic (cycling)	12-13 RPE	3	6 months	NR	8
Huang et al. (2014)	Taiwan	NRT	66	Modified HIT (First 12 sessions MICT and then 24 HIT sessions at)	VT for MICT, 7x3 min HIT intervals at 80% VO2 reserve	3	8 weeks	NR	8
Tasoulis et al. (2010)	Greece	RCT	57	Aerobic interval training with or without ST	50% of Steep Ramp Test and ST at 55-65% 2RM	3	12 weeks	NR	8
Parnell et al. (2005)	Australia	RCT	21	Aerobic (walking, light hand-weights and cycling)	50-60% MHR	3/5-7 (3 supervised, encouraged to perform 5-7 times home-based)	8 weeks	NR	7
Mandic et al. (2009)	New Zealand	RCT	42	Aerobic (treadmill and cycling)	50-70% HRR	3	12 weeks	0 (one AF episode during training)	11
				Aerobic (treadmill and cycling) and resistance (dynamic)	50-70% 1-RM				
Servantes et al. (2018)	Brazil	RCT	37	Aerobic (treadmill and cycling)	HR corresponding to AT	3	3 months	0	11

Koukouvou et al. (2004)	Greece	RCT	26	Aerobic (cycling, walking/jogging, stair climber, step-aerobics), calisthenics, and resistance training added >3 months	50-70% peak VO2 and RPE 12-14	3-4	6 months	0	11
Dalal et al. (2019)	UK	RCT	216	Chair based exercise or progressive walking	No metric	≥3	4 months	0	12
Piotrowicz et al. (2020)	Poland	RCT	845	Aerobic (walking), respiratory muscle training and resistance (resistance band)	40-70% HRR, 30-60% max inspiratory mouth pressure, no metric for RT	Everyday	9 weeks	0	14
Myers et al. (2007)	Switzerland	RCT	24	Aerobic (walking and cycling)	60-80% HRR for cycling	Walking every day, 4 cycling sessions	2 months	NR	8
O'Connor et al. (2009)	USA, Canada and France	RCT	2331	Aerobic (walking, treadmill or cycling)	60-70% HRR	3 supervised, then 5 home	3 months	37 hospitalizations, 5 deaths	14
Flynn et al. (2009)	USA, Canada and France	RCT	2331	Aerobic (walking, treadmill or cycling)	60-70% HRR	3 supervised, then 5 home	3 months	37 hospitalizations, 5 deaths	11
Ahmad et al. (2014)	USA, Canada and France	RCT	928	Aerobic (walking, treadmill or cycling)	60-70% HRR	3 supervised, then 5 home	3 months	37 hospitalizations, 5 deaths	11
Witham et al. (2012)	UK	RCT	107	Intermittent functional aerobic exercise training and strength training	No Metric	2	24 weeks	1 self-limiting angina episode and 1 fall	12
Sandri et al. (2012)	Germany	RCT	60	Aerobic (cycling) (<55 years of age)	70% symptom limited peak VO2	4	4 weeks	0	10
				Aerobic (cycling) (>65 years of age)	70% symptom limited peak VO2				

AT= Anaerobic threshold, HRR= Heart rate reserve, MHR= Maximal heart rate, RCP= Respiratory compensation point, RPE= rate perceived exertion, VT= Ventilatory threshold, Wrpeak= Work rate peak, 1-RM= 1 repetition maximu

Statistical analysis:

Note: Insufficient data to perform any HFpEF sub-group analysis (on RCT vs NRT, Supervised vs Unsupervised, and Exercise type).

Exercise Capacity:

Table S5: HFpEF peak VO_2 moderator analysis

Moderator	Coefficient	P-value
Baseline VO_2	-0.1294	0.2684
Age	-0.2123	0.2339
Sex	0.0430	0.2155
BMI	0.2035	0.3872
NYHA class	0.0081	0.6348
Intervention duration (weeks)	-0.1160	0.4524
Study design (RCT vs NRT)	Insufficient Data	Insufficient Data
Ischemic Etiology	Insufficient Data	Insufficient Data
Dilated Etiology	Insufficient Data	Insufficient Data
Hypertensive Etiology	Insufficient Data	Insufficient Data

Table S6: HFrEF peak VO_2 moderator analysis

Moderator	Coefficient	P-value
Baseline VO_2	0.0609	0.4849
Age	-0.0984	0.0059*
Sex	-0.0183	0.2273
BMI	0.0050	0.9798
NYHA class	-0.0102	0.3271
Intervention duration (weeks)	0.0061	0.8634
Study design (RCT vs NRT)	0.6746	0.3113
Ischemic Etiology	-0.0021	0.8825
Dilated Etiology	-0.0056	0.6837
Hypertensive Etiology	0.0972	0.0121*

Figure S1: Age Peak VO2 HFrEF regression bubble plot

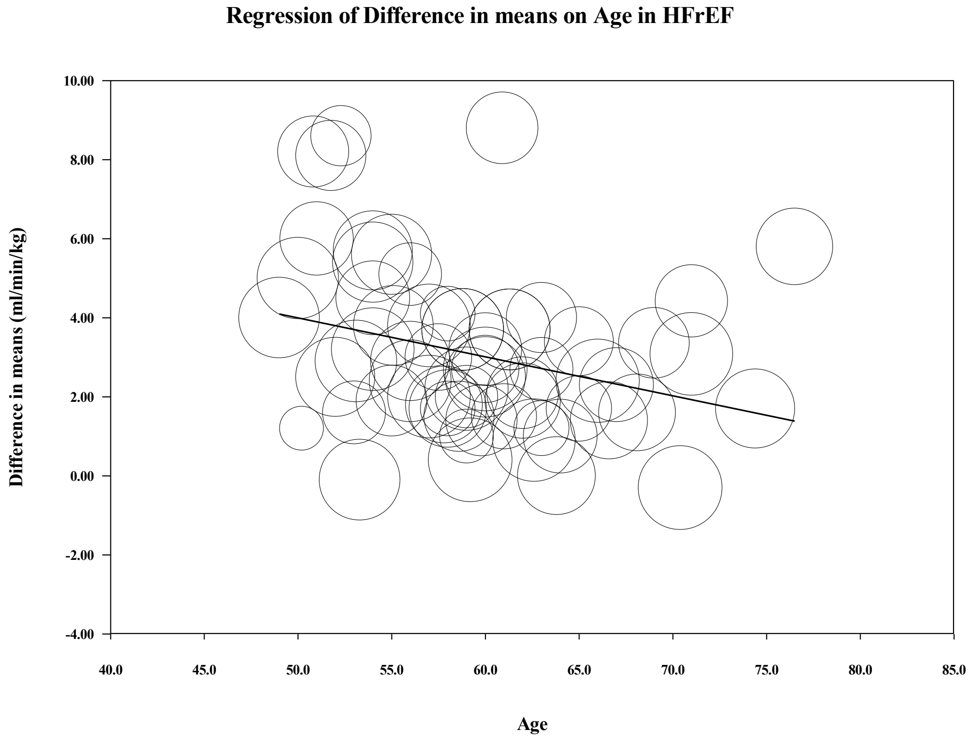

Figure S2: Hypertensive HF Etiology Peak VO2 HFrEF regression bubble plot

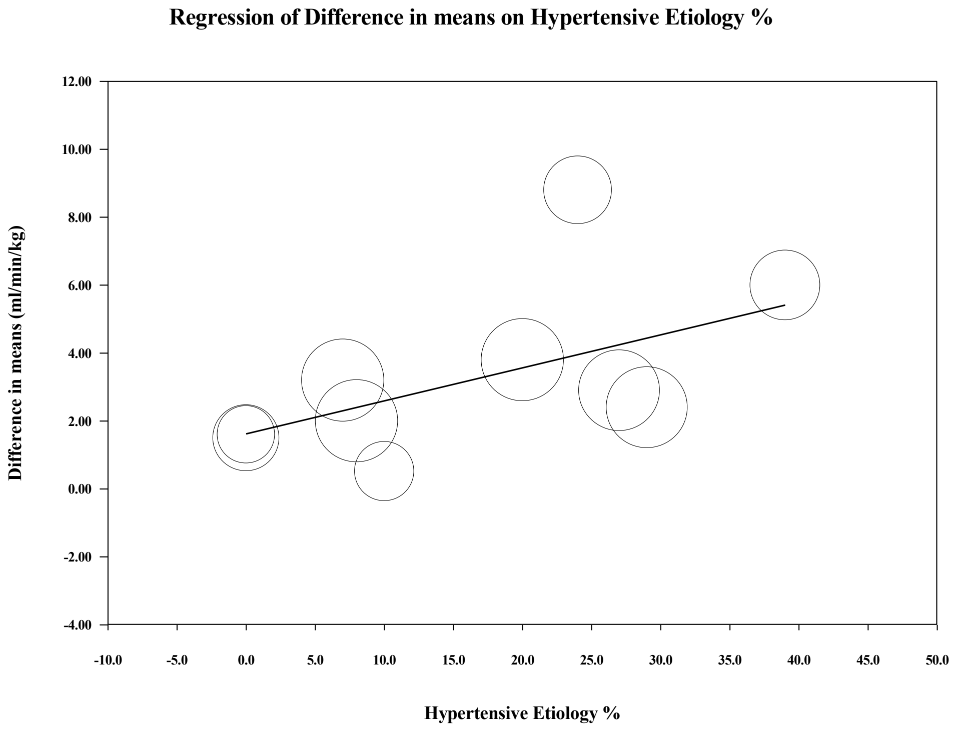

Quality of Life:

Table S7: HFpEF MLHFQ moderator analysis

Moderator	Coefficient	P-value
Baseline MLHFQ	-0.7386	0.0026*
Age	0.4418	0.5099
Sex	-0.1672	0.3103
BMI	0.0259	0.9872
NYHA class	-0.0001	0.9992
Intervention duration (weeks)	1.2791	0.0672

Figure S3: Baseline MLHFQ HFpEF regression bubble plot.

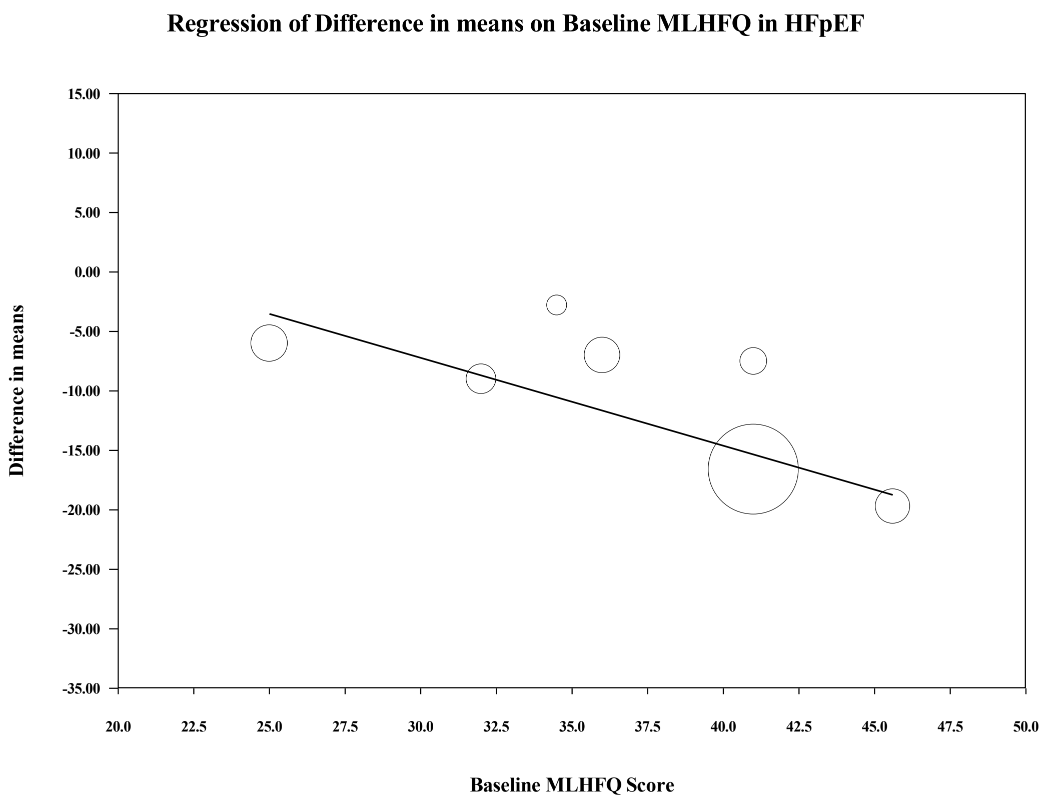

Table S8: HFrEF MLHFQ moderator analysis

Moderator	Coefficient	P-value
Baseline MLHFQ	-0.2852	0.0283*
Age	0.4820	0.1151
Sex	0.3006	0.0012*
BMI	0.1208	0.9182
NYHA class	0.1279	0.0872
Intervention duration (weeks)	0.2909	0.2575
Study design (RCT vs NRT)	6.3334	0.0511
Ischemic Etiology	0.1290	0.0972
Dilated Etiology	0.1205	0.2141
Hypertensive Etiology	-0.4452	0.0751

Figure S4: Baseline MLHFQ HFrEF regression bubble plot

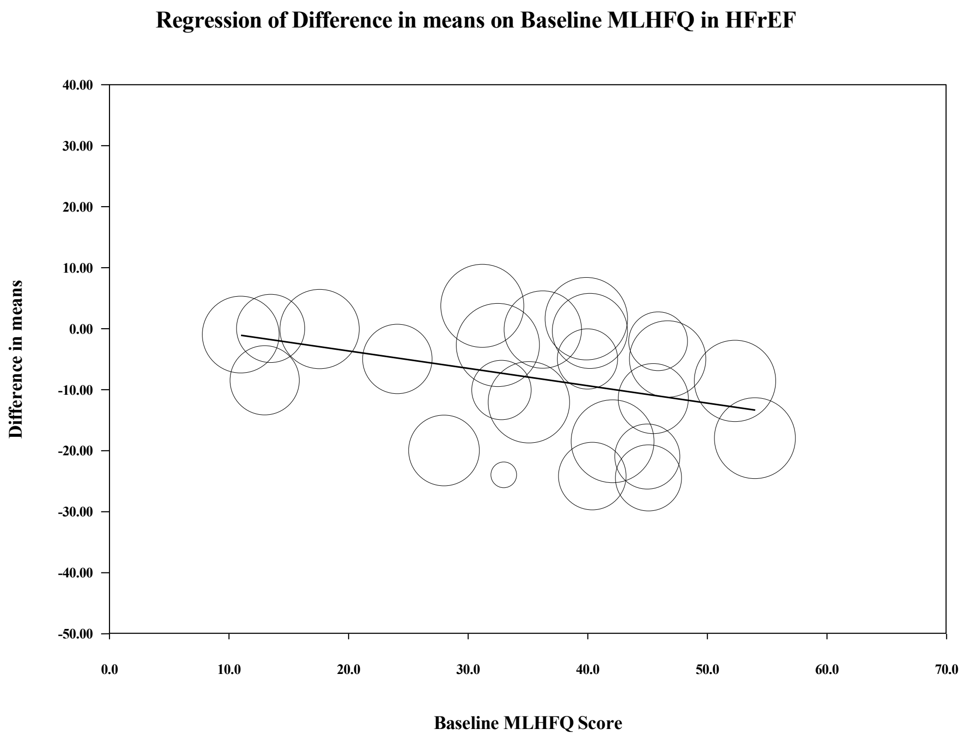

Figure S5: Gender MLHFQ HFrEF regression bubble plot

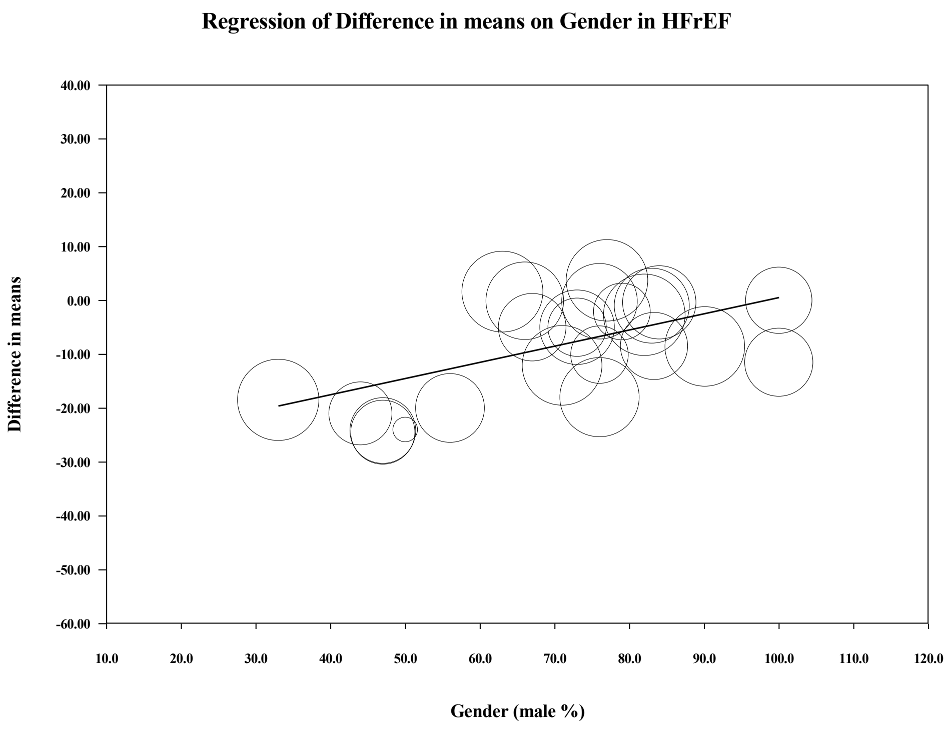

Cardiac function

Table S9: HFpEF LVEF moderator analysis

Moderator	Coefficient	P-value
Baseline LVEF	-0.2448	0.3194
Age	-0.1130	0.6415
Sex	0.0520	0.4109
BMI	-0.1224	0.6458
NYHA class	-0.0385	0.3806
Intervention duration (weeks)	-0.1390	0.6192

Table S10: HFrEF LVEF moderator analysis

Moderator	Coefficient	P-value
Baseline LVEF	-0.0998	0.6616
Age	-0.0132	0.9294
Sex	-0.0936	0.0911
BMI	0.1612	0.6428
NYHA class	0.0592	0.0394*
Intervention duration (weeks)	-0.1482	0.3606
Study design (RCT vs NRT)	-1.8654	0.3266
Ischemic Etiology	0.0350	0.2940
Dilated Etiology	-0.0231	0.5719
Hypertensive Etiology	0.0685	0.4224

Figure S6: NYHA Class LVEF HFrEF regression bubble plot.

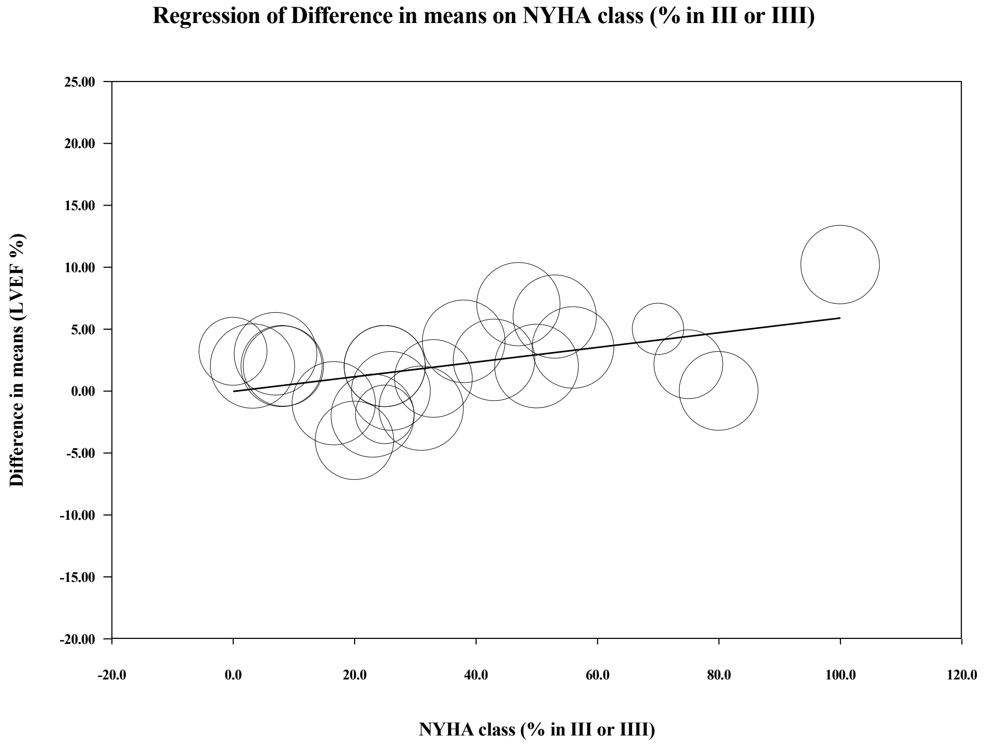

BNP

Table S11: HFrEF BNP moderator analysis

Moderator	Coefficient	P-value
Age	0.2010	0.1235
Sex	-0.1114	0.0404*
BMI	Insufficient Data	Insufficient Data
NYHA class	-0.0233	0.4130
Intervention duration (weeks)	0.2237	0.0054*
Study design (RCT vs NRT)	-0.8338	0.3832
Ischemic Etiology	-0.0614	0.2114
Dilated Etiology	Insufficient Data	Insufficient Data
Hypertensive Etiology	Insufficient Data	Insufficient Data

Figure S7: Sex BNP HFrEF regression bubble plot.

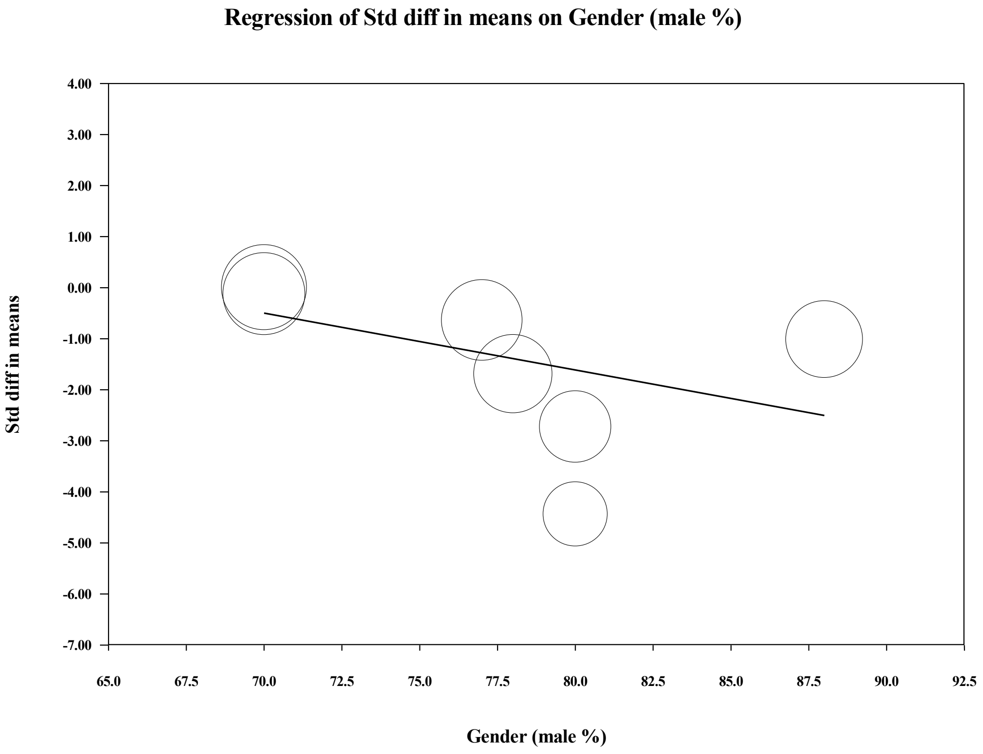

Figure S8: Intervention duration BNP HFrEF regression bubble plot.

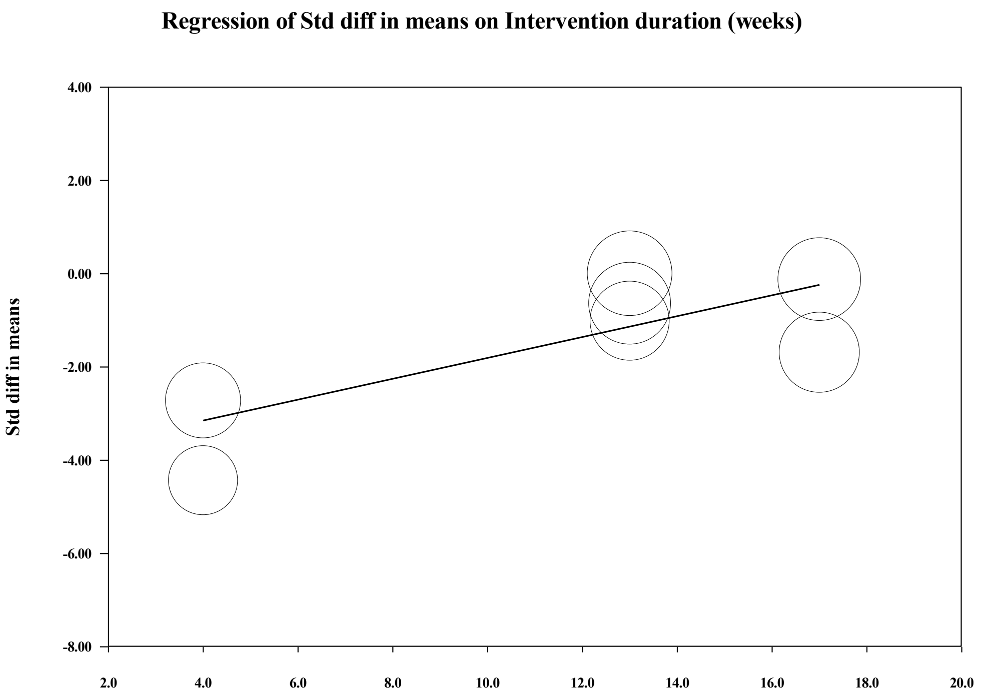

Analysed studies

HFpEF:

1. Alves AJ, Ribeiro F, Goldhammer E, Rivlin Y, Rosenschein U, Viana JL, Duarte JA, Sagiv M, Oliveira J. Exercise training improves diastolic function in heart failure patients. Med Sci Sports Exerc. 2012;44:776–785
2. Edelmann, F., Gelbrich, G., Düngen, H.D., Fröhling, S., Wachter, R., Stahrenberg, R., Binder, L., Töpper, A., Lashki, D.J., Schwarz, S. and Herrmann-Lingen, C., 2011. Exercise training improves exercise capacity and diastolic function in patients with heart failure with preserved ejection fraction: results of the Ex-DHF (Exercise training in Diastolic Heart Failure) pilot study. Journal of the American College of Cardiology, 58(17), pp.1780-1791.
3. Fu, T.C., Yang, N.I., Wang, C.H., Cherng, W.J., Chou, S.L., Pan, T.L. and Wang, J.S., 2016. Aerobic interval training elicits different hemodynamic adaptations between heart failure patients with preserved and reduced ejection fraction. American journal of physical medicine & rehabilitation, 95(1), pp.15-27.
4. Gary, R.A., Sueta, C.A., Dougherty, M., Rosenberg, B., Cheek, D., Preisser, J., Neelon, V. and McMurray, R., 2004. Home-based exercise improves functional performance and quality of life in women with diastolic heart failure. Heart & Lung, 33(4), pp.210-218.
5. Karavidas A, Driva M, Parissis JT, Farmakis D, Mantzaraki V, Varounis C, Paraskevaidis I, Ikonomidis I, Pirgakis V, AnastasiouNana M, Filippatos G. Functional electrical stimulation of peripheral muscles improves endothelial function and clinical and emotional status in heart failure patients with preserved left ventricular ejection fraction. Am Heart J 166: 760–767, 2013.
6. Kitzman DW, Brubaker PH, Morgan TM, Haykowsky M, Hundley G, Kraus WE, Eggebeen J, Nicklas BJ (2016) Effect of caloric restriction or aerobic exercise training on peak oxygen consumption and quality of life in obese older patients with heart failure with preserved ejection fraction: a randomized clinical trial. JAMA 315(1):36–46. https://doi.org/10.1001/jama.2015.17346
7. Kitzman, D.W., Brubaker, P.H., Herrington, D.M., Morgan, T.M., Stewart, K.P., Hundley, W.G., Abdelhamed, A. and Haykowsky, M.J., 2013. Effect of endurance exercise training on endothelial function and arterial stiffness in older patients with heart failure and preserved ejection fraction: a randomized, controlled, single-blind trial. Journal of the American college of cardiology, 62(7), pp.584-592.
8. Kitzman, D.W., Brubaker, P.H., Morgan, T.M., Stewart, K.P. and Little, W.C., 2010. Exercise training in older patients with heart failure and preserved ejection fraction: a randomized, controlled, single-blind trial. Circulation: Heart Failure, 3(6), pp.659-667.
9. Mueller, S., Winzer, E.B., Duvinage, A., Gevaert, A.B., Edelmann, F., Haller, B., Pieske-Kraigher, E., Beckers, P., Bobenko, A., Hommel, J. and Van de Heyning, C.M., 2021. Effect of high-intensity interval training, moderate continuous training, or guideline-based physical activity advice on peak oxygen consumption in patients with heart failure with preserved ejection fraction: a randomized clinical trial. Jama, 325(6), pp.542-551.
10. Palau P, Dominguez E, Nunez E, Schmid JP, Vergara P, Ramón JM, Mascarell B, Sanchis J, Chorro FJ, Núñez J. Effects of inspiratory muscle training in patients with heart failure with preserved ejection fraction. Eur J Prev Cardiol 21: 1465–1473, 2014.
11. Smart, N.A., Haluska, B., Jeffriess, L. and Leung, D., 2012. Exercise training in heart failure with preserved systolic function: a randomized controlled trial of the effects on cardiac function and functional capacity. Congestive heart failure, 18(6), pp.295-301.

HFrEF:

1. Acanfora, D., Scicchitano, P., Casucci, G., Lanzillo, B., Capuano, N., Furgi, G., Acanfora, C., Longobardi, M., Incalzi, R.A., Piscosquito, G. and Ciccone, M.M., 2016. Exercise training effects on elderly and middle-age patients with chronic heart failure after acute decompensation: A randomized, controlled trial. International journal of cardiology, 225, pp.313-323.
2. Ahmad, T., Fiuzat, M., Mark, D.B., Neely, B., Neely, M., Kraus, W.E., Kitzman, D.W., Whellan, D.J., Donahue, M., Zannad, F. and Piña, I.L., 2014. The effects of exercise on cardiovascular biomarkers in patients with chronic heart failure. American heart journal, 167(2), pp.193-202.
3. Antunes-Correa, L.M., Melo, R.C., Nobre, T.S., Ueno, L.M., Franco, F.G., Braga, A.M., Rondon, M.U., Brum, P.C., Barretto, A.C., Middlekauff, H.R. and Negrao, C.E., 2010. Impact of gender on benefits of exercise training on sympathetic nerve activity and muscle blood flow in heart failure. European journal of heart failure, 12(1), pp.58-65.
4. Beckers, P.J., Denollet, J., Possemiers, N.M., Wuyts, K., Vrints, C.J. and Conraads, V.M., 2010. Maintaining physical fitness of patients with chronic heart failure: a randomized controlled trial. European Journal of Preventive Cardiology, 17(6), pp.660-667.
5. Belardinelli, R., Capestro, F., Misiani, A., Scipione, P. and Georgiou, D., 2006. Moderate exercise training improves functional capacity, quality of life, and endothelium-dependent vasodilation in chronic heart failure patients with implantable cardioverter defibrillators and cardiac resynchronization therapy. European Journal of Preventive Cardiology, 13(5), pp.818-825.
6. Belardinelli, R., Georgiou, D., Cianci, G. and Purcaro, A., 1999. Randomized, controlled trial of long-term moderate exercise training in chronic heart failure: effects on functional capacity, quality of life, and clinical outcome. Circulation, 99(9), pp.1173-1182.
7. Belardinelli, R., Georgiou, D., Scocco, V., Barstow, T.J. and Purcaro, A., 1995. Low intensity exercise training in patients with chronic heart failure. Journal of the American College of Cardiology, 26(4), pp.975-982.
8. Brubaker, P.H., Moore, J.B., Stewart, K.P., Wesley, D.J. and Kitzman, D.W., 2009. Endurance exercise training in older patients with heart failure: results from a randomized, controlled, single-blind trial. Journal of the American Geriatrics Society, 57(11), pp.1982-1989.
9. Chien, C.L., Lee, C.M., Wu, Y.W. and Wu, Y.T., 2011. Home-based exercise improves the quality of life and physical function but not the psychological status of people with chronic heart failure: a randomised trial. Journal of physiotherapy, 57(3), pp.157-163.
10. Chou, C.H., Fu, T.C., Tsai, H.H., Hsu, C.C., Wang, C.H. and Wang, J.S., 2019. High-intensity interval training enhances mitochondrial bioenergetics of platelets in patients with heart failure. International journal of cardiology, 274, pp.214-220.
11. Chrysohoou, C., Angelis, A., Tsitsinakis, G., Spetsioti, S., Nasis, I., Tsiachris, D., Rapakoulias, P., Pitsavos, C., Koulouris, N.G., Vogiatzis, I. and Dimitris, T., 2015. Cardiovascular effects of high-intensity interval aerobic training combined with strength exercise in patients with chronic heart failure. A randomized phase III clinical trial. International journal of cardiology, 179, pp.269-274.

12. Conraads, V.M., Beckers, P., Vaes, J., Martin, M., Van Hoof, V., De Maeyer, C., Possemiers, N., Wuyts, F.L. and Vrints, C.J., 2004. Combined endurance/resistance training reduces NT-proBNP levels in patients with chronic heart failure. European heart journal, 25(20), pp.1797-1805.
13. Corvera-Tindel, T., Doering, L.V., Woo, M.A., Khan, S. and Dracup, K., 2004. Effects of a home walking exercise program on functional status and symptoms in heart failure. American heart journal, 147(2), pp.339-346.
14. Dalal, H.M., Taylor, R.S., Jolly, K., Davis, R.C., Doherty, P., Miles, J., Van Lingen, R., Warren, F.C., Green, C., Wingham, J. and Greaves, C., 2019. The effects and costs of home-based rehabilitation for heart failure with reduced ejection fraction: The REACH-HF multicentre randomized controlled trial. European journal of preventive cardiology, 26(3), pp.262-272.
15. de Mello Franco, F.G., Santos, A.C., Rondon, M.U.P., Trombetta, I.C., Strunz, C., Braga, A.M.W., Middlekauff, H., Negrao, C.E. and Barretto, A.C.P., 2006. Effects of home-based exercise training on neurovascular control in patients with heart failure. European journal of heart failure, 8(8), pp.851-855.
16. Demopoulos, L., Yeh, M., Gentilucci, M., Testa, M., Bijou, R., Katz, S.D., Mancini, D., Jones, M. and LeJemtel, T.H., 1997. Nonselective β-adrenergic blockade with carvedilol does not hinder the benefits of exercise training in patients with congestive heart failure. Circulation, 95(7), pp.1764-1767.
17. Dracup, K., Evangelista, L.S., Hamilton, M.A., Erickson, V., Hage, A., Moriguchi, J., Canary, C., MacLellan, W.R. and Fonarow, G.C., 2007. Effects of a home-based exercise program on clinical outcomes in heart failure. American heart journal, 154(5), pp.877-883.
18. Du, H., Newton, P.J., Budhathoki, C., Everett, B., Salamonson, Y., Macdonald, P.S. and Davidson, P.M., 2018. The Home-Heart-Walk study, a self-administered walk test on perceived physical functioning, and self-care behaviour in people with stable chronic heart failure: A randomized controlled trial. European Journal of Cardiovascular Nursing, 17(3), pp.235-245.
19. Eleuteri, E., Mezzani, A., Di Stefano, A., Vallese, D., Gnemmi, I., Delle Donne, L., Taddeo, A., Della Bella, S. and Giannuzzi, P., 2013. Aerobic training and angiogenesis activation in patients with stable chronic heart failure: a preliminary report. Biomarkers, 18(5), pp.418-424.
20. Erbs, S., Höllriegel, R., Linke, A., Beck, E.B., Adams, V., Gielen, S., Möbius-Winkler, S., Sandri, M., Kränkel, N., Hambrecht, R. and Schuler, G., 2010. Exercise training in patients with advanced chronic heart failure (NYHA IIIb) promotes restoration of peripheral vasomotor function, induction of endogenous regeneration, and improvement of left ventricular function. Circulation: Heart Failure, 3(4), pp.486-494.
21. Fayazi, S., Zarea, K., Abbasi, A. and Ahmadi, F., 2013. Effect of home-based walking on performance and quality of life in patients with heart failure. Scandinavian journal of caring sciences, 27(2), pp.246-252.
22. Flynn, K.E., Piña, I.L., Whellan, D.J., Lin, L., Blumenthal, J.A., Ellis, S.J., Fine, L.J., Howlett, J.G., Keteyian, S.J., Kitzman, D.W. and Kraus, W.E., 2009. Effects of exercise training on health status in patients with chronic heart failure: HF-ACTION randomized controlled trial. Jama, 301(14), pp.1451-1459.

23. Fraga, R., Franco, F.G., Roveda, F., de Matos, L.N., Braga, A.M., Rondon, M.U., Rotta, D.R., Brum, P.C., Barretto, A.C., Middlekauff, H.R. and Negrão, C.E., 2007. Exercise training reduces sympathetic nerve activity in heart failure patients treated with carvedilol. European journal of heart failure, 9(6-7), pp.630-636.
24. Freimark, D., Shechter, M., Schwamenthal, E., Tanne, D., Elmaleh, E., Shemesh, Y., Motro, M. and Adler, Y., 2007. Improved exercise tolerance and cardiac function in severe chronic heart failure patients undergoing a supervised exercise program. International journal of cardiology, 116(3), pp.309-314.
25. Fu, T.C., Yang, N.I., Wang, C.H., Cherng, W.J., Chou, S.L., Pan, T.L. and Wang, J.S., 2016. Aerobic interval training elicits different hemodynamic adaptations between heart failure patients with preserved and reduced ejection fraction. American journal of physical medicine & rehabilitation, 95(1), pp.15-27.
26. Gademan, M.G., Swenne, C.A., Verwey, H.F., Van de Vooren, H., Haest, J.C., van Exel, H.J., Lucas, C.M., Cleuren, G.V., Schalij, M.J. and Van der Wall, E.E., 2008. Exercise training increases oxygen uptake efficiency slope in chronic heart failure. European Journal of Preventive Cardiology, 15(2), pp.140-144.
27. Gary, R.A., Cress, M.E., Higgins, M.K., Smith, A.L. and Dunbar, S.B., 2012. A combined aerobic and resistance exercise program improves physical functional performance in patients with heart failure: a pilot study. The Journal of cardiovascular nursing, 27(5), p.418.
28. Giannuzzi, P., Temporelli, P.L., Corrà, U. and Tavazzi, L., 2003. Antiremodeling effect of long-term exercise training in patients with stable chronic heart failure: results of the Exercise in Left Ventricular Dysfunction and Chronic Heart Failure (ELVD-CHF) Trial. Circulation, 108(5), pp.554-559.
29. Gielen, S., Adams, V., Möbius-Winkler, S., Linke, A., Erbs, S., Yu, J., Kempf, W., Schubert, A., Schuler, G. and Hambrecht, R., 2003. Anti-inflammatory effects of exercise training in the skeletal muscle of patients with chronic heart failure. Journal of the American College of Cardiology, 42(5), pp.861-868.
30. Gottlieb, S.S., Fisher, M.L., Freudenberger, R., Robinson, S., Zietowski, G., Alves, L., Krichten, C., Vaitkevicus, P. and Mccarter, R., 1999. Effects of exercise training on peak performance and quality of life in congestive heart failure patients. Journal of cardiac failure, 5(3), pp.188-194.
31. Groehs, R.V., Toschi-Dias, E., Antunes-Correa, L.M., Trevizan, P.F., Rondon, M.U.P., Oliveira, P., Alves, M.J., Almeida, D.R., Middlekauff, H.R. and Negrão, C.E., 2015. Exercise training prevents the deterioration in the arterial baroreflex control of sympathetic nerve activity in chronic heart failure patients. American Journal of Physiology-Heart and Circulatory Physiology, 308(9), pp.H1096-H1102.
32. Groennebaek, T., Sieljacks, P., Nielsen, R., Pryds, K., Jespersen, N.R., Wang, J., Carlsen, C.R., Schmidt, M.R., de Paoli, F.V., Miller, B.F. and Vissing, K., 2019. Effect of blood flow restricted resistance exercise and remote ischemic conditioning on functional capacity and myocellular adaptations in patients with heart failure. Circulation: Heart Failure, 12(12), p.e006427.
33. Hambrecht, R., Fiehn, E., Weigl, C., Gielen, S., Hamann, C., Kaiser, R., Yu, J., Adams, V., Niebauer, J. and Schuler, G., 1998. Regular physical exercise corrects endothelial dysfunction and improves exercise capacity in patients with chronic heart failure. circulation, 98(24), pp.2709-2715.

34. Hambrecht, R., Gielen, S., Linke, A., Fiehn, E., Yu, J., Walther, C., Schoene, N. and Schuler, G., 2000. Effects of exercise training on left ventricular function and peripheral resistance in patients with chronic heart failure: a randomized trial. Jama, 283(23), pp.3095-3101.
35. Harjola, V.P., Kiilavuori, K. and Virkamäki, A., 2006. The effect of moderate exercise training on skeletal muscle myosin heavy chain distribution in chronic heart failure. International journal of cardiology, 109(3), pp.335-338.
36. Höllriegel, R., Winzer, E.B., Linke, A., Adams, V., Mangner, N., Sandri, M., Bowen, T.S., Hambrecht, R., Schuler, G. and Erbs, S., 2016. Long-Term Exercise Training in Patients With Advanced Chronic Heart Failure. Journal of cardiopulmonary rehabilitation and prevention, 36(2), pp.117-124.
37. Huang, S.C., Wong, M.K., Lin, P.J., Tsai, F.C., Fu, T.C., Wen, M.S., Kuo, C.T. and Wang, J.S., 2014. Modified high-intensity interval training increases peak cardiac power output in patients with heart failure. European journal of applied physiology, 114(9), pp.1853-1862.
38. Isaksen, K., Munk, P.S., Valborgland, T. and Larsen, A.I., 2015. Aerobic interval training in patients with heart failure and an implantable cardioverter defibrillator: a controlled study evaluating feasibility and effect. European journal of preventive cardiology, 22(3), pp.296-303.
39. Jolly, K., Taylor, R.S., Lip, G.Y., Davies, M., Davis, R., Mant, J., Singh, S., Greenfield, S., Ingram, J., Stubley, J. and Bryan, S., 2009. A randomized trial of the addition of home-based exercise to specialist heart failure nurse care: the Birmingham Rehabilitation Uptake Maximisation study for patients with Congestive Heart Failure (BRUM-CHF) study. European journal of heart failure, 11(2), pp.205-213.
40. Kemps, H.M., De Vries, W.R., Schmikli, S.L., Zonderland, M.L., Hoogeveen, A.R., Thijssen, E.J. and Schep, G., 2010. Assessment of the effects of physical training in patients with chronic heart failure: the utility of effort-independent exercise variables. European journal of applied physiology, 108(3), pp.469-476.
41. Kiilavuori, K., Sovijärvi, A., Näveri, H., Ikonen, T. and Leinonen, H., 1996. Effect of physical training on exercise capacity and gas exchange in patients with chronic heart failure. Chest, 110(4), pp.985-991.
42. Kobayashi, N., Tsuruya, Y., Iwasawa, T., Ikeda, N., Hashimoto, S., Yasu, T., Ueba, H., Kubo, N., Fujii, M., Kawakami, M. and Saito, M., 2003. Exercise training in patients with chronic heart failure improves endothelial function predominantly in the trained extremities. Circulation journal, 67(6), pp.505-510.
43. Koukouvou, G., Kouidi, E., Iacovides, A., Konstantinidou, E., Kaprinis, G. and Deligiannis, A., 2004. Quality of life, psychological and physiological changes following exercise training in patients with chronic heart failure. Journal of rehabilitation medicine, 36(1), pp.36-41.
44. Lan, N.S., Lam, K., Naylor, L.H., Green, D.J., Minaee, N.S., Dias, P. and Maiorana, A.J., 2020. The Impact of Distinct Exercise Training Modalities on Echocardiographic Measurements in Patients with Heart Failure with Reduced Ejection Fraction. Journal of the American Society of Echocardiography, 33(2), pp.148-156.
45. Maiorana, A.J., Naylor, L.H., Exterkate, A., Swart, A., Thijssen, D.H., Lam, K., O'Driscoll, G. and Green, D.J., 2011. The impact of exercise training on conduit artery

wall thickness and remodeling in chronic heart failure patients. Hypertension, 57(1), pp.56-62.
46. Mandic, S., Tymchak, W., Kim, D., Daub, B., Quinney, H.A., Taylor, D., Al-Kurtass, S. and Haykowsky, M.J., 2009. Effects of aerobic or aerobic and resistance training on cardiorespiratory and skeletal muscle function in heart failure: a randomized controlled pilot trial. Clinical rehabilitation, 23(3), pp.207-216.
47. McKelvie, R.S., Teo, K.K., Roberts, R., McCartney, N., Humen, D., Montague, T., Hendrican, K. and Yusuf, S., 2002. Effects of exercise training in patients with heart failure: the Exercise Rehabilitation Trial (EXERT). American heart journal, 144(1), pp.23-30.
48. Meirelles, L.R.D., Matsuura, C., Resende, A.D.C., Salgado, Â.A., Pereira, N.R., Coscarelli, P.G., Mendes-Ribeiro, A.C. and Brunini, T.M., 2014. Chronic exercise leads to antiaggregant, antioxidant and anti-inflammatory effects in heart failure patients. European journal of preventive cardiology, 21(10), pp.1225-1232.
49. Myers, J., Hadley, D., Oswald, U., Bruner, K., Kottman, W., Hsu, L. and Dubach, P., 2007. Effects of exercise training on heart rate recovery in patients with chronic heart failure. American heart journal, 153(6), pp.1056-1063.
50. Nishi, I., Noguchi, T., Iwanaga, Y., Furuichi, S., Aihara, N., Takaki, H. and Goto, Y., 2011. Effects of exercise training in patients with chronic heart failure and advanced left ventricular systolic dysfunction receiving β-blockers. Circulation Journal, pp.1105171230-1105171230.
51. Nobre, T.S., Antunes-Correa, L.M., Groehs, R.V., Alves, M.J.N., Sarmento, A.O., Bacurau, A.V., Urias, U., Alves, G.B., Rondon, M.U.P., Brum, P.C. and Martinelli, M., 2016. Exercise training improves neurovascular control and calcium cycling gene expression in patients with heart failure with cardiac resynchronization therapy. American Journal of Physiology-Heart and Circulatory Physiology, 311(5), pp.H1180-H1188.
52. O'Connor, C.M., Whellan, D.J., Lee, K.L., Keteyian, S.J., Cooper, L.S., Ellis, S.J., Leifer, E.S., Kraus, W.E., Kitzman, D.W., Blumenthal, J.A. and Rendall, D.S., 2009. Efficacy and safety of exercise training in patients with chronic heart failure: HF-ACTION randomized controlled trial. Jama, 301(14), pp.1439-1450.
53. Oka, R.K., De Marco, T., Haskell, W.L., Botvinick, E., Dae, M.W., Bolen, K. and Chatterjee, K., 2000. Impact of a home-based walking and resistance training program on quality of life in patients with heart failure. The American journal of cardiology, 85(3), pp.365-369.
54. Palevo, G., Keteyian, S.J., Kang, M. and Caputo, J.L., 2009. Resistance exercise training improves heart function and physical fitness in stable patients with heart failure. Journal of cardiopulmonary rehabilitation and prevention, 29(5), pp.294-298.
55. Parnell, M.M., Holst, D.P. and Kaye, D.M., 2002. Exercise training increases arterial compliance in patients with congestive heart failure. Clinical science, 102(1), pp.1-7.
56. Parnell, M.M., Holst, D.P. and Kaye, D.M., 2005. Augmentation of endothelial function following exercise training is associated with increased L-arginine transport in human heart failure. Clinical Science, 109(6), pp.523-530.
57. Piotrowicz, E., Pencina, M.J., Opolski, G., Zaręba, W., Banach, M., Kowalik, I., Orzechowski, P., Szalewska, D., Pluta, S., Główczyńska, R. and Irzmański, R., 2020. Effects of a 9-week hybrid comprehensive telerehabilitation program on long-term

outcomes in patients with heart failure: the Telerehabilitation in Heart Failure Patients (TELEREH-HF) randomized clinical trial. JAMA cardiology, 5(3), pp.300-308.

58. Pozehl, B., Duncan, K., Hertzog, M. and Norman, J.F., 2010. Heart failure exercise and training camp: effects of a multicomponent exercise training intervention in patients with heart failure. Heart & Lung, 39(6), pp.S1-S13.
59. Ricca-Mallada, R., Migliaro, E.R., Silvera, G., Chiappella, L., Frattini, R. and Ferrando-Castagnetto, F., 2017. Functional outcome in chronic heart failure after exercise training: Possible predictive value of heart rate variability. Annals of physical and rehabilitation medicine, 60(2), pp.87-94.
60. Roveda, F., Middlekauff, H.R., Rondon, M.U.P., Reis, S.F., Souza, M., Nastari, L., Barretto, A.C.P., Krieger, E.M. and Negrão, C.E., 2003. The effects of exercise training on sympathetic neural activation in advanced heart failure: a randomized controlled trial. Journal of the American College of Cardiology, 42(5), pp.854-860.
61. Safiyari-Hafizi, H., Taunton, J., Ignaszewski, A. and Warburton, D.E., 2016. The health benefits of a 12-week home-based interval training cardiac rehabilitation program in patients with heart failure. Canadian Journal of Cardiology, 32(4), pp.561-567.
62. Sandri, M., Kozarez, I., Adams, V., Mangner, N., Höllriegel, R., Erbs, S., Linke, A., Möbius-Winkler, S., Thiery, J., Kratzsch, J. and Teupser, D., 2012. Age-related effects of exercise training on diastolic function in heart failure with reduced ejection fraction: the Leipzig Exercise Intervention in Chronic Heart Failure and Aging (LEICA) Diastolic Dysfunction Study. European heart journal, 33(14), pp.1758-1768.
63. Sarullo, F.M., Gristina, T., Brusca, I., Milia, S., Raimondi, R., Sajeva, M., La Chiusa, S.M., Serio, G., Paterna, S., Di Pasquale, P. and Castello, A., 2006. Effect of physical training on exercise capacity, gas exchange and N-terminal pro-brain natriuretic peptide levels in patients with chronic heart failure. European Journal of Preventive Cardiology, 13(5), pp.812-817.
64. Selig, S.E., Carey, M.F., Menzies, D.G., Patterson, J., Geerling, R.H., Williams, A.D., Bamroongsuk, V., Toia, D., Krum, H. and Hare, D.L., 2004. Moderate-intensity resistance exercise training in patients with chronic heart failure improves strength, endurance, heart rate variability, and forearm blood flow. Journal of cardiac failure, 10(1), pp.21-30.
65. Senden, P.J., Sabelis, L.W., Zonderland, M.L., Hulzebos, E.H., Bol, E. and Mosterd, W.L., 2005. The effect of physical training on workload, upper leg muscle function and muscle areas in patients with chronic heart failure. International journal of cardiology, 100(2), pp.293-300.
66. Servantes, D.M., Javaheri, S., Kravchychyn, A.C.P., Storti, L.J., Almeida, D.R., de Mello, M.T., Cintra, F.D., Tufik, S. and Bittencourt, L., 2018. Effects of exercise training and CPAP in patients with heart failure and OSA: a preliminary study. Chest, 154(4), pp.808-817.
67. Servantes, D.M., Pelcerman, A., Salvetti, X.M., Salles, A.F., de Albuquerque, P.F., de Salles, F.C.A., Lopes, C., de Mello, M.T., Almeida, D.R. and Filho, J.A.O., 2012. Effects of home-based exercise training for patients with chronic heart failure and sleep apnoea: a randomized comparison of two different programmes. Clinical rehabilitation, 26(1), pp.45-57.
68. Spee, R.F., Niemeijer, V.M., Wijn, P.F., Doevendans, P.A. and Kemps, H.M., 2016. Effects of high-intensity interval training on central haemodynamics and skeletal muscle

oxygenation during exercise in patients with chronic heart failure. European journal of preventive cardiology, 23(18), pp.1943-1952.

69. Stevens, A.L., Hansen, D., Herbots, L., Wens, I., Creemers, A., Dendale, P. and Eijnde, B.O., 2015. Exercise training improves insulin release during glucose tolerance testing in stable chronic heart failure patients. Journal of cardiopulmonary rehabilitation and prevention, 35(1), pp.37-46.
70. Sturm, B., Quittan, M., Wiesinger, G.F., Stanek, B., Frey, B. and Pacher, R., 1999. Moderate-intensity exercise training with elements of step aerobics in patients with severe chronic heart failure. Archives of physical medicine and rehabilitation, 80(7), pp.746-750.
71. Tasoulis, A., Papazachou, O., Dimopoulos, S., Gerovasili, V., Karatzanos, E., Kyprianou, T., Drakos, S., Anastasiou-Nana, M., Roussos, C. and Nanas, S., 2010. Effects of interval exercise training on respiratory drive in patients with chronic heart failure. Respiratory medicine, 104(10), pp.1557-1565.
72. Testa, M., Ennezat, P.V., Vikstrom, K.L., Demopoulos, L., Gentilucci, M., Loperfido, F., Fanelli, R., Kitsis, R.N., Leinwand, L.A. and LeJemtel, T.H., 2000. Modulation of vascular endothelial gene expression by physical training in patients with chronic heart failure. Italian Heart Journal, 1, pp.426-430.
73. Tyni-Lenné, R., Dencker, K., Gordon, A., Jansson, E. and Sylvén, C., 2001. Comprehensive local muscle training increases aerobic working capacity and quality of life and decreases neurohormonal activation in patients with chronic heart failure. European Journal of Heart Failure, 3(1), pp.47-52.
74. Tyni-Lenné, R., Gordon, A. and Sylvén, C., 1996. Improved quality of life inchronic heart failure patients following local endurance training with leg muscles. Journal of cardiac failure, 2(2), pp.111-117.
75. Van Berendoncks, A.M., Beckers, P., Hoymans, V.Y., Possemiers, N., Wuyts, F.L., Vrints, C.J. and Conraads, V.M., 2010. Exercise training reduces circulating adiponectin levels in patients with chronic heart failure. Clinical science, 118(4), pp.281-289.
76. van den Berg-Emons, R., Balk, A., Bussmann, H. and Stam, H., 2004. Does aerobic training lead to a more active lifestyle and improved quality of life in patients with chronic heart failure?. European Journal of Heart Failure, 6(1), pp.95-100.
77. Willenheimer, R., Erhardt, L., Cline, C., Rydberg, E. and Israelsson, B., 1998. Exercise training in heart failure improves quality of life and exercise capacity. European heart journal, 19(5), pp.774-781.
78. Wisløff, U., Støylen, A., Loennechen, J.P., Bruvold, M., Rognmo, Ø., Haram, P.M., Tjønna, A.E., Helgerud, J., Slørdahl, S.A., Lee, S.J. and Videm, V., 2007. Superior cardiovascular effect of aerobic interval training versus moderate continuous training in heart failure patients: a randomized study. Circulation, 115(24), pp.3086-3094.
79. Witham, M.D., Fulton, R.L., Greig, C.A., Johnston, D.W., Lang, C.C., van der Pol, M., Boyers, D., Struthers, A.D. and McMurdo, M.E., 2012. Efficacy and cost of an exercise program for functionally impaired older patients with heart failure: a randomized controlled trial. Circulation: Heart Failure, 5(2), pp.209-216.
80. Witham, M.D., Gray, J.M., Argo, I.S., Johnston, D.W., Struthers, A.D. and McMurdo, M.E., 2005. Effect of a seated exercise program to improve physical function and health status in frail patients≥ 70 years of age with heart failure. The American journal of cardiology, 95(9), pp.1120-1124.

81. Yeh, G.Y., McCarthy, E.P., Wayne, P.M., Stevenson, L.W., Wood, M.J., Forman, D., Davis, R.B. and Phillips, R.S., 2011. Tai chi exercise in patients with chronic heart failure: a randomized clinical trial. Archives of internal medicine, 171(8), pp.750-757.
82. Zurek, M., Corrà, U., Piepoli, M.F., Binder, R.K., Saner, H. and Schmid, J.P., 2012. Exercise training reverses exertional oscillatory ventilation in heart failure patients. European respiratory journal, 40(5), pp.1238-1244.
